# Evaluation of geometric tortuosity for 3D digitally generated porous media considering the pore size distribution and the A-star algorithm

**DOI:** 10.1038/s41598-022-23643-6

**Published:** 2022-11-14

**Authors:** Joseph Ávila, Javier Pagalo, Mayken Espinoza-Andaluz

**Affiliations:** 1 Facultad de Ingeniería en Electricidad y Computación, Escuela Superior Politécnica del Litoral, ESPOL, Campus Gustavo Galindo Km. 30.5 Vía Perimetral, P.O. Box 09-01-5863, Guayaquil, Ecuador; 2 Facultad de Ingeniería Mecánica y Ciencias de la Producción, Escuela Superior Politécnica del Litoral, ESPOL, Campus Gustavo Galindo Km. 30.5 Vía Perimetral, P.O. Box 09-01-5863, Guayaquil, Ecuador; 3 Facultad de Ingeniería Mecánica y Ciencias de la Producción, Centro de Energías Renovables y Alternativas, Escuela Superior Politécnica del Litoral, ESPOL, Campus Gustavo Galindo Km. 30.5 Vía Perimetral, P.O. Box 09-01-5863, Guayaquil, Ecuador

**Keywords:** Mechanical engineering, Hydrology, Computational science

## Abstract

Porous materials are of great interest in multiple applications due to their usefulness in energy conversion devices and their ability to modify structural and diffusive properties. Geometric tortuosity plays an important role in characterizing the complexity of a porous medium. The literature on several occasions has related it as a parameter dependent on porosity only. However, due to its direct relationship with the morphology of the medium, a deeper analysis is necessary. For this reason, in the present study, the analysis of the geometric tortuosity is proposed considering the porosity and the pore size distribution. Geometric tortuosity in artificially generated digital porous media is estimated using the A-star algorithm and the Pore Centroid method. By performing changes in the size of the medium and the distribution of the pore size, results are obtained that indicate that the geometric tortuosity does not only depend on the porosity. By maintaining the same porosity, the geometric tortuosity increases if the pore size is reduced. Similarly, these pore size effects are greater if the size of the medium is reduced. The A-star algorithm was found to be more suitable to characterize the majority of paths within the half-pore. On the other hand, to increase the size, the Pore Centroid method is the most appropriate. Finally, three types of correlations were generated relating tortuosity with porosity and pore size. All the correlations were determined with 95% of interval confidence.

## Introduction

Porous materials are composed of two phases, i.e., solid and pore volumes, distributed disorderly^1^, resulting in complex media. Porous media are constituted by a solid skeleton (matrix) and the pore spaces (pore network)^2^. Following the International Union of Pure and Applied Chemistry (IUPAC), porous materials can be classified into three categories according to their pore diameter: microporous (< 2 nm), mesoporous (2—50 nm), and macroporous (> 50 nm) materials^3^.

Some microstructural descriptors let to characterize the porous media. Porosity, specific surface area, correlation functions, pore size distribution, Minkowski functionals, constrictivity, and tortuosity are essential parameters to describe porous materials^4,5^. Tortuosity is a singular parameter used to characterize the flow paths inside a porous medium^6^, giving an idea of the medium's complexity and quantifying the flux resistance of the structure. Tortuosity is commonly correlated with porosity^5,6^, but it is also related to other parameters depending on the described phenomena^6^. There are several types of tortuosity: electrical, hydraulic, diffusive, and geometrical^5,6^. Geometric tortuosity emphasizes the microstructure configuration, focusing on the geometric flow paths inside the media^6^, in contrast with the other types of flux-based tortuosity^5^. Geometric tortuosity is valuable for different areas, considering that changes in the microstructure of electrochemical devices can affect their performance^7^. In electrochemistry, it has been useful for the microstructural characterization of lithium-ion batteries^8^ as well as porous support layers^9^. Even a correlation between geometric and flux-based tortuosity for LiFePO4 electrodes^10^ has been developed. For earth sciences, it provides a reliable estimation of pathways for 3D rock porous media samples as well as can be used to estimate other parameters such as permeability and effective diffusion coefficient helping in applications such as gas storage and reservoir production^11^. Additionally, for durability evaluations of porous composites such as fuel cell electrodes, filtration membranes, polymer foams, ceramics, and powder beds, geometric tortuosity is important^12^.

The mathematical definition of geometric tortuosity is as follows^6^:

1 $${\tau }_{geometric}=\frac{{L}_{g}}{L}$$

where $L$ is the shortest route length of the porous media and ${L}_{g}$ is the mean of the lengths of the effective routes. The parameter *L* is just the length of the straight line between the inlet and outlet surface of the analyzed medium.

Modeling allows the generation of porous media with a great variety of microstructures and different complexities. Voxel-based models are particularly interesting because they are directly linked with the experimental data collected from volumetric images^13^. Moreover, it lets a better analysis of its geometrical properties.

This study focusses on the analysis of digitally generated artificial porous media rather than real porous media obtained by tomography. This methodology allows the reduction of expenses by not needing to acquire expensive equipment for the processing of porous materials. Instead, only computational power would be required for analysis using simulation techniques. Furthermore, the simulation methods are non-destructive, unlike other processes that require the application of forces or pressure for analysis, such as mercury porosimetry or gas absorption processes.

There are well-known programs that allow the generation of artificial porous media with specific properties allowing the control of multiple geometric parameters of the medium^14,15^. This reduces the differences between digitally generated media and real media, allowing similar results in both methodologies. As a result, generating artificial media digitally allows correlations to be obtained, since it is easier to experiment with a large number of samples that would be difficult to achieve in reality depending on the type of media to be studied.

There are numerous ways to generate explicit microstructure models: overlapping of spheres^16^, Voronoi tessellations^1^, random packing of simple solids^4^, or imitating physical process methods^17^. Considering the several methodology generations, different software has been employed to analyze the generated porous media. PuMA^18^, Tort3D^19^, OpenPNM^15^, and Porespy^14^ are some of the computational tools to generate and study porous materials from different approaches.

This study is focused on the computation of geometrical tortuosity on 3D artificially generated porous media. Porespy, a Python toolkit for microstructure study of porous media^14^, has been used to carry out the current research. The porous media are generated using the Gaussian blur method. Similar studies have been performed with some modifications related to processing and generating methods^20–22^. One of the main analyzed parameters in the current study is the geometric tortuosity based on Eq. (1). According to the definition, all the possible route lengths through the porous media have to be identified to compute the geometric tortuosity. These routes correspond to the trajectories through which the species would pass. In the context of transport phenomena, it would be where the fluid passes or where the mass transfer occurs. In general, tortuosity is related to the resistance to the flow of matter that a structure has. The more paths there are for the passage of species, the less resistance there will be. In the same way, the fewer paths there are, the resistance would increase. This would also depend on the porosity of the medium, the size of the pores that make up this path, or the distribution of grains, in summary, the microstructure. The geometric tortuosity is usually considered due to its direct relationship with the geometry of the porous medium since it can estimate a resistance value of the medium without defining a particular specie. Given the nature of this type of tortuosity and its direct relation to paths in the porous medium, pathfinding algorithms are widely used. Algorithms such as the direct shortest-path search^23^, the skeleton shortest path^24^, and the Dijkstra algorithm^25^ can be applied to estimate geometric tortuosity. Other algorithms such as pore centroid^7^ and fast marching^8^ are also used for geometric tortuosity estimation.

In the current study, the possible paths are determined by applying path-search and image-based algorithms. The A-star algorithm on 3D spaces is used to complete the pathfinding approach, while the pore centroid is used to estimate involved nodes. The advantage that the A-star algorithm has over the other algorithms presented is the heuristic function that allows it to evaluate the most optimal neighbor based on its proximity to the target. This heuristic function works during the route estimation process, reducing the computational cost that could occur if it were only evaluated by random neighbors until reaching the destination. Although the A-star algorithm is not the only path-search algorithm, and some methods can pre-process the map to guarantee efficiencies such as contraction hierarchies^26^ and transit routes^27^, previous findings have demonstrated that A-star is a powerful tool and a well-known best-first search algorithm due to its wide application range in solving problems^28,29^. In addition, the A-star algorithm has also been used to estimate geometric tortuosity in 3D porous media^11^.

Previous studies correlate tortuosity uniquely with porosity^30,31^. A well-known correlation is the Bruggeman equation, expressed as an exponential equation^6^. Based mainly on random packs of grains arrangement, this correlation depends on an empirical constant called Bruggeman's constant. Several studies reviewed by Tjaden et al.^7^ have shown a partial validity of this correlation for the calculation of tortuosity in real media. Fu et al.^5^ present the Bruggeman correlation and other tortuosity-porosity correlations in their review article and mentioned the fact that the present correlations in the theory depend only on porosity. Considering that a porous medium in the real world depends on several microstructural characteristics that often come from irregular geometries, an adequate value of tortuosity would not be reached only the porosity is considered. As demonstrated in the computational research presented by Espinoza-Andaluz et al.^32^, tortuosity depends not only on the effective porosity but also on the particle shape of the media. The effect of the morphological characteristics on porous media properties is also analyzed by other studies^33–35^. In addition, an important feature of the existing correlations in the theory is that most consider isotropic porous media, with regular particle arrangements, to be valid for a limited number of models. For general use where a larger range of real media is considered, an analysis of heterogeneous porous media is adequate. This is possible as long as an arrangement of particles in random locations and sizes can be guaranteed. Due to this, to approach more realistic forms, this study proposes the analysis of porous media generated with a random array. To perform a comparison with the existing correlations in the literature, tortuosity correlations will be generated as a function of porosity but varying the pore size distribution. Finally, to ensure that a realistic characterization is done, a correlation of tortuosity as a function of the average pore size is presented.

The Gaussian blur method is applied to generate the porous media in which the porosity and the standard deviation of the Gaussian kernel are needed. The standard deviation controls the morphology modifying the grain arrangement and as a result the pore size distribution. The current study proposes three kinds of correlations: a tortuosity-porosity correlation, a correlation that relates the tortuosity with the porosity and the pore size distribution which depends on the Gaussian kernel´s standard deviation, and a correlation of tortuosity with the average pore size solely. To the best author's knowledge, there is no report of the standard deviation impact on the pore size of generated Gaussian porous media. There are reports about porous media generation methodologies that consider the Gaussian blur method without considering the standard deviation of the Gaussian kernel^36–38^. The mentioned standard deviation plays an essential role in this approach since it affects the internal morphology of the medium by controlling the amount of blurring (more details in “Porous media generation”). Therefore, the second proposed correlation can be used when the Gaussian blur method is applied. For better presentation, the standard deviation of the Gaussian kernel will be called as typical, sigma.

As part of the study, pore size distribution (PSD) is also computed. The pore size distribution can be defined as a measure of abundance for each existing pore size in a volume of each porous medium. It can be represented as a function of the pore radius, whose values vary in every part of the mentioned volume^39^. The frequency of pore sizes represents a pore size distribution when it expresses equivalent pore diameters developing a continuous function^40^. Empirically, PSD can be obtained by the two well-known methods, gas adsorption and porosimetry of mercury, which uses pressure with fluid invasion^41^. However, given the current study's computational nature, the PSD is determined using Porespy through the pore size distribution function (PSF).

In studies where porous media are reconstructed, the representative elementary volume (REV) is initially defined^22,42^. Since the current research considers digitally created porous media, the bulk porosity of media is known from the beginning. Therefore, it is intended to define specific sizes and porosities, assuming that these values will not change over time. The volume from which the REV starts will be obtained from an artificially generated medium of larger dimensions and intermediate microstructural characteristics than our study.

On the other hand, porous media samples are generated based on random images, assuming they represent porous media, and tortuosity is estimated. This study seeks to test the A-star algorithm as an alternative to tortuosity evaluation as well as to review the implications of its use. As mentioned, and cited earlier in this section, tortuosity evaluation has been carried out with path-finding algorithms in various situations, even with different particle shapes.

The rest of the paper is divided as follows: “Methodology” explains the applied methodology, porous media generation, and geometric tortuosity computation. In addition, the pore size distribution approach is described. Results and discussions are presented in “Results and discussions”. Tortuosity correlations as a function of the bulk porosity and the effect of the pore size distribution are presented and compared to previous studies. Finally, conclusions and future directions are presented in “Conclusions”.

## Methodology

This section explains the methodology for solving paths through the porous media with the A-star search algorithm. Before the A-star application, the variables included in the study are defined, and the generation process of the porous media are explained.

### Porous media generation

The porous media creation starts with a matrix image of random noise generated with zeros and ones. Then, a multidimensional Gaussian filter is applied. It is implemented as a sequence of 1-D Gaussian filters as follows:

2 $$G\left(x\right)=\frac{1}{\sqrt{2\pi }\sigma } {e}^{-\frac{{x}^{2} }{2{\sigma }^{2}}}$$

where the variable $x$ is dependent on the sigma of the kernel, it covers a range of values as follows:

3 $$- int\left(4\cdot \sigma + 0.5\right)<x< int\left(4\cdot \sigma + 0.5\right)+1$$

where $int()$ is a function that returns an integer. Furthermore, the sigma of the kernel is equal to the mean of the image's size divided by 40 times $\delta ,$ which is a correction parameter named blobiness. Being ${N}_{x}, {N}_{y}$ and ${N}_{z}$ the sizes of the tridimensional generated image, the sigma value results as a function of the blobiness parameter is computed as follows:

4 $$\sigma (\delta )=\frac{\frac{{N}_{x}+{N}_{y}+{N}_{z}}{3}}{40\cdot \delta }$$

The sigma formulation is intended to allow images with visually pleasing solid and porous space distribution at low blobiness values^14^. The blobiness is the variable that controls the morphology of the porous media and falls in the 0 to 1 range. By reducing its value, the standard deviation of the Gaussian Filter increases, and larger pore diameters on the final structure are expected.

A Gaussian convolution is applied to the defined kernel and previously generated random image. The result is a normally distributed image converted to a uniform (flat) distribution. Therefore, the resulting image can be defined as a grey-scale matrix ${\psi }_{ijk}\left(x\right)$. The solid–pore interface is set up by defining a threshold, *ψ*_0_, which falls in the range from zero to one. All pixel values in the last resulting image greater than the threshold are rounded to the value one and defined as the solid phase. In contrast, a value of 0 is defined as the pore phase, resulting in a set of binary values ${\psi }_{\left(bin\right)ijk}$ as in Carvalho et al.^42^. Therefore, the pore indicator function is written as:

5 $$\chi \left(x\right) := \left\{\begin{array}{c} \\ \begin{array}{cc}1& \mathrm{ if }{\psi }_{ijk}\left(x\right)\ge {\psi }_{0}\\ 0& \mathrm{if }{\psi }_{ijk}\left(x\right)< {\psi }_{0}\end{array}\end{array}\right.$$

where $\chi \left(x\right)$ is used to convert the last resulting image's grey-scale matrix into a 0/1 binary matrix, the edge level ${\psi }_{0}$, in the current study, is equal to the porosity. On the other hand, the two-dimensional porosity $\phi$ can be defined as the pore area divided by the total area. In two-dimensional images, it can be obtained by counting the number of pixels in the solid phase and dividing by the total number of pixels from the binarized images. Therefore, the porosity is written as^42^:

6 $$\phi =1-\frac{\sum_{i=1}^{{N}_{x}}\sum_{j=1}^{{N}_{y}}\sum_{k=1}^{{N}_{z}}{\psi }_{\left(bin\right)ijk}}{{N}_{x}\mathrm{x}{N}_{y}\mathrm{x}{N}_{z}}$$

The following steps describe, in summary, the algorithm to generate the porous media:

1. An image of random noise is generated. Its size is defined by the shape parameter. It is a Python matrix filled with numbers between 0 and 1.
2. A Gaussian kernel of sigma determined by the mean of shape parameter dividing by a factor is generated. The mentioned factor is a number chosen such that a blobiness of 1 gave reasonable-sized blobs^14^. It controls the size and aspect ratio of the solid particles.
3. Gaussian blur convolution is applied over the image of step 1. This process is performed by using the defined kernel.
4. The resulting image is normalized and rescaled with values between 0 and 1.
5. Thresholding is applied over the image until the value of specified bulk porosity is reached.

**Figure 1:**
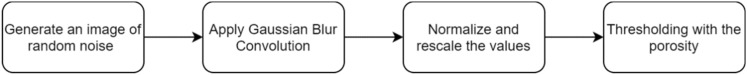
Flowchart of porous media generation process with corresponding sub-processes.

A simplified description of the porous media generation process is presented in Fig. 1. Four main sub-processes are depicted.

**Figure 2:**
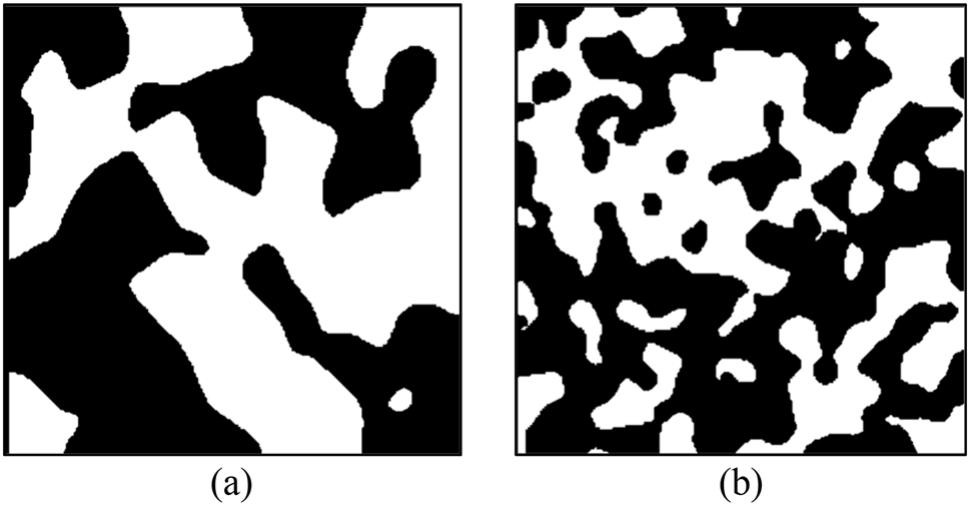
Cross-sectional view of the samples, obtained by Espinoza et al.^43^, with the same porosity (ε = 0.5) and different blobiness. The left side (**a**) corresponds to a porous media with blobiness of 0.5, while the right (**b**) represents a porous media with blobiness equal to 1.0.

To analyze the effect of the blobiness over the generated porous media, Fig. 2 compares two 2D porous media with different blobiness values where the black color represents solids and white represents void spaces. The current study evaluates the blobiness value in the 0.5–1 range.

### Porous media selection

A binary 3D matrix represents porous media, so each voxel is characterized by either a zero or one. The solid material is represented by one, while the void spaces are considered zeros. The mentioned approach is commonly used in artificial porous media computation^5^. The size domain of the porous media is given in voxels, which result in the tridimensional representation of pixels. Considering an image resolution of 1 µm per pixel as well as other studies that also use Porespy as the generator of porous media^44–46^, it can be concluded that every voxel represents 1 µm^3^ in size. The more voxels the porous media has, the more resolution of the geometrical properties can be achieved. However, an analysis of porous media with high dimensions requires high computational power. Therefore, the current study's size domain is a cubic volume with 40 × 40 × 40 pixels or 40^3^ voxels, representing a porous media of 40^3^ µm^3^.

**Figure 3:**
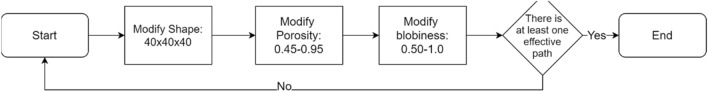
The flowchart shows the porous media generation steps considering the porosity and blobiness values^43^.

Six different values of blobiness are evaluated, from 0.5 to 1.0, with a leap of 0.10. For each blobiness, eleven different porosities are analyzed, i.e., from 0.45 to 0.95 in steps of 0.05. In the end, a total of 3300 samples were generated. The number of samples is determined considering statistical approaches to guarantee the appropriateness of the results (see Online Appendix A). On the other hand, the porosity values were selected based on practical applications in several fields: biomaterials (0.70–0.95)^47^, metal foams (0.75–0.95)^48^, gas diffusion layers (0.60–0.90)^31^, Lithium-Ion battery electrodes (0.30–0.60)^49^, catalyst layers (0.40–0.60)^31^ and porous media in other fuel cells (0.30–0.55)^50^. Figure 3 shows the flowchart describing how the porous media are generated, considering the bulk porosity and blobiness.

To generate a medium, it is necessary to establish the mold media represented by the volume. It determines how many nodes occupy the three-dimensional space. Considering the computational cost, size-independent analysis (see Online Appendix B), and Representative Elementary Volume analysis (see Online Appendix C) a cubic volume with forty elements by side has been selected to represent the porous media.

Then, once the shape is defined, it is required to establish a porosity range to study. In this step, it is important to consider the more common porosities in porous media applications. The porosity range is determined considering the previously mentioned materials, so the porosity range is from 0.45 to 0.95. The blobiness value is modified and varied from 0.5 to 1.0 to represent a wide variety of materials in which the morphological structure of the solid-pore interaction has effects. With the variables established, a medium is generated for the Porespy library. All the generated domains are just candidates because it is needed to ensure at least one possible effective path between the inlet and outlet surfaces. If no connection is possible, the geometric tortuosity cannot be computed. Porous media with at least an effective route are stored as stereolithography CAD in a NumPy array. Paraview is used to open the stereolithography CAD and report a better image of the generated porous media.

### Path selection

Since the generated media are 40 × 40 × 40 shape, to estimate the number of paths the number of inlet ${p}_{in}$ and outlet ${p}_{out}$ nodes are defined, and the number of paths is computed as ${p}_{in}*{p}_{out}$. Considering the direction of flow as the z-axis, in the case that both inlet and outlet surfaces were all void nodes, ${p}_{in}*{p}_{out}$ will be 1600*1600, therefore the number of possible paths to compute will be $2.56{*10}^{6}$ paths. Although the number of possible paths would be reduced depending on the porous media connectivity, the computational resources are still occupied as the algorithm must validate every possible path. To avoid the aforementioned case, it is proposed to reduce the number of possible paths from the reduction of the number of inlet ${p}_{in}$ and outlet ${p}_{out}$ nodes through a porous medium cutting algorithm. The general idea of this process is to cut both the inlet and outlet surfaces into 100 parts each and to obtain the possible node as the midpoint of each cut if it exists. After this procedure, in the case that exists a midpoint in every cut, the number of inlet ${p}_{in}$ and outlet ${p}_{out}$ nodes are reduced to 100, obtaining a number of possible paths to compute equal to $1{*10}^{4}$ reducing the computational cost. To obtain better details of this process, the following procedure is followed:

- Slice the porous media layer into 100 parts
- Apply the pore centroid approach
- Apply A-star algorithm

#### Matrix division

**Figure 4:**
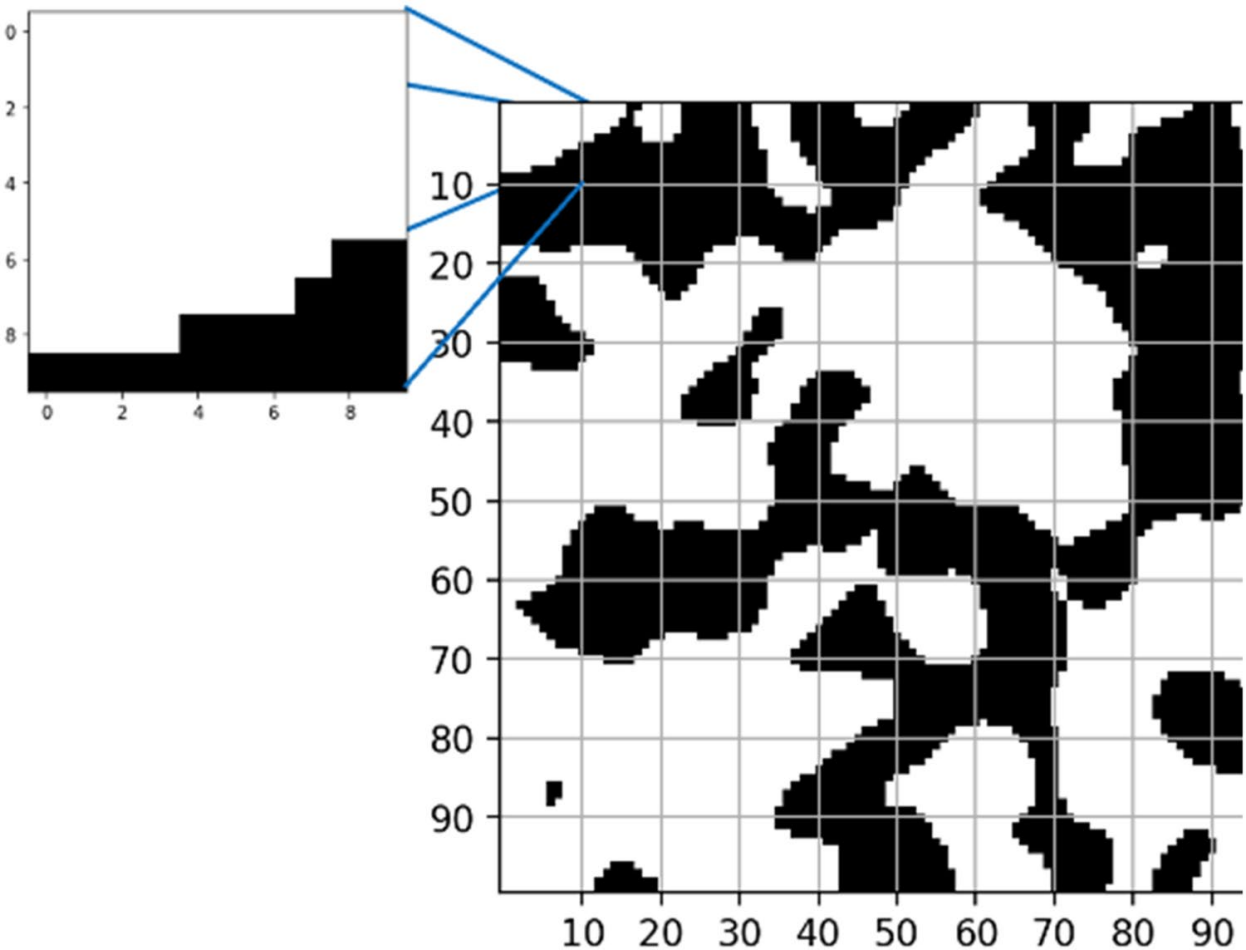
Sectioned regions of the entrance slice to analyze the possible inlet of the paths found within the domain. The inlet surface (100 × 100) has been divided into 10 × 10 subregions.

The first approach is implemented to reduce the computational cost of considering all the initial and final nodes that form the porous media paths. The process takes the matrix representation of the porous media previously saved in the porous media generation process. The first layer of the matrix is taken, considering that the z-axis indicates the depth of the 3D porous media. Then, the layer represented by z = 1 is selected and separated into smaller slices. Figure 4 presents a graphical explanation of the sub-division for one selected slice. The process is repeated until the last layer, z = L, where L is the length of the porous media on the z-axis. Layers z = 1 and z = L will allow us to find the input and output paths.

#### Pore centroid approach

**Figure 5:**
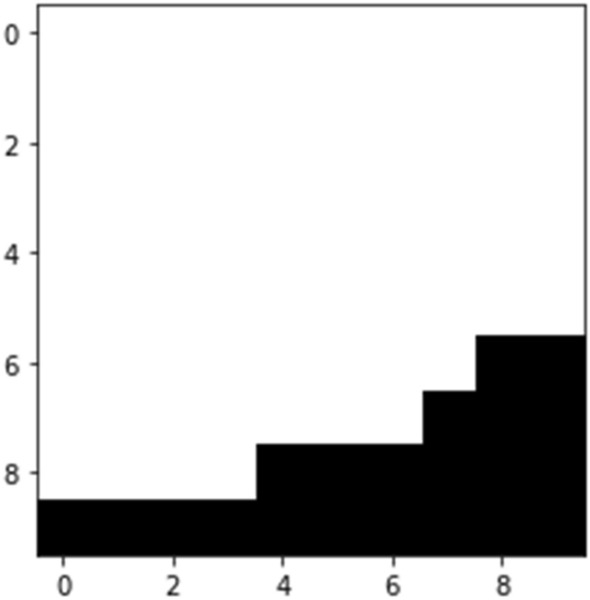
First sectioned region to analyze the position of the inlet path.

**Figure 6:**
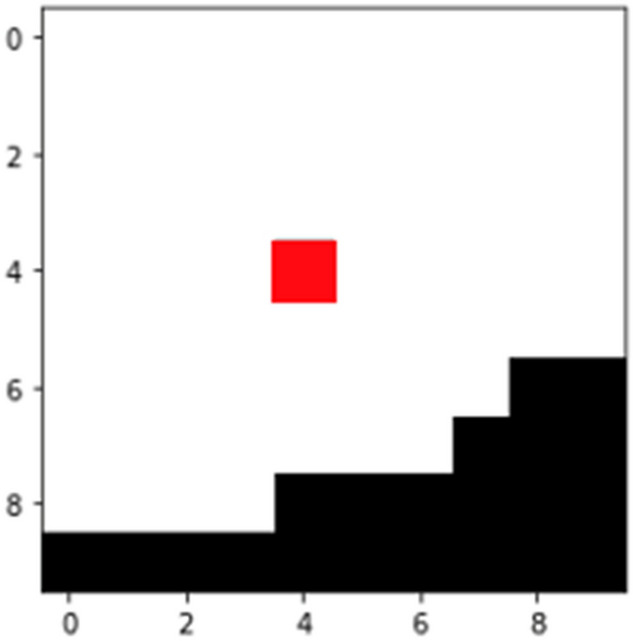
Position of the inlet path based on the center of mass of the void surface.

The pore centroid of every sub-region in layers z = 1 and z = L are determined once the Matrix Division procedure is carried out. The objective of the pore centroid calculations is to get the possible initial and final nodes that will form the path that allows the computation of the geometric tortuosity^7^. The current study only uses the pore centroid approach to determine the pore centroid nodes. Following the mentioned procedure, 100 paths at maximum are guaranteed to reduce the computational cost. Figures 5 and 6 show the graphical representation of the followed process on a 10 × 10 slice.

**Figure 7:**
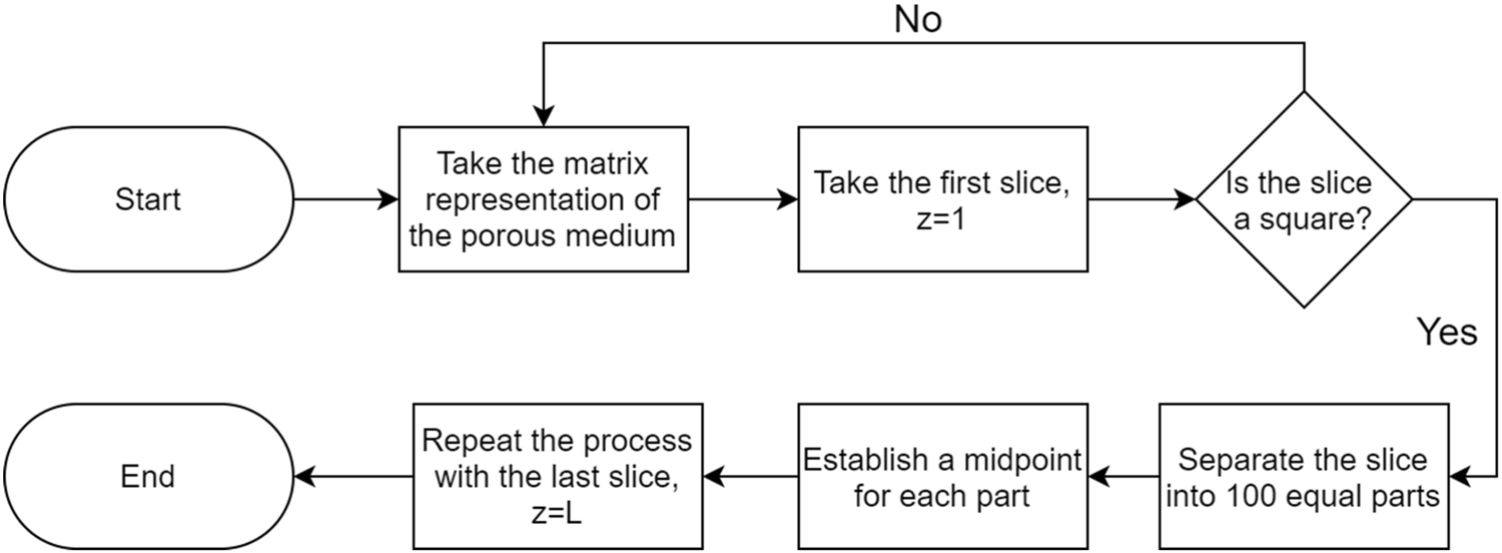
Flowchart showing the steps for the path selection.

Matrix division and the selection of the midpoint processes are resumed in the following flowchart in Fig. 7.

#### A-star algorithm

The A-Star algorithm belongs to the best-known path search algorithms widely applied in a space with a metric or topological configuration^46^. The algorithm uses a heuristic search and search-based methods on the shortest route. The main objective of the algorithm is to find a path from a selected starting point to an endpoint. Both points or nodes are obtained with the Pore Centroid approximation considering the lowest cost, i.e., the shortest route traveled. Analyzed nodes can be freely located in the space or arranged in the form of a structured grid. In the current study, given the binary geometry, the void nodes serve as a computational space for the A-Star algorithm. In such a case, the path can pass only through the nodes with a value representing the pore space. One limitation is that a route cannot pass twice through the same node on the network. The A-Star algorithm is defined as a best-first algorithm because the value evaluates each cell in the configuration space as follows:

7 $$f\left(x,y,z\right)=h\left(x,y,z\right)+g(x,y,z)$$

where *h(x, y, z)* is the heuristic distance from the cell to the desired state, *g(x, y, z)* is the path length from the initial state to the desired state according to the selected series of cells. This list ends with the scheduled cell. The value *f(x, y, z)* evaluates each cell adjacent to the target cell. The cell with the lowest value, *f(x, y, z),* is selected as the next cell in the sequence. The Euclidean distance is used to determine the distance between two nodes. It is used to value the nodes in the heuristic formula, and the following equation gives it:

8 $$d\left({P}_{1},{P}_{2}\right)=\sqrt{{({x}_{1}-{x}_{2})}^{2}+{({y}_{1}-{y}_{2})}^{2}+{({z}_{1}-{z}_{2})}^{2}}$$

**Figure 8:**
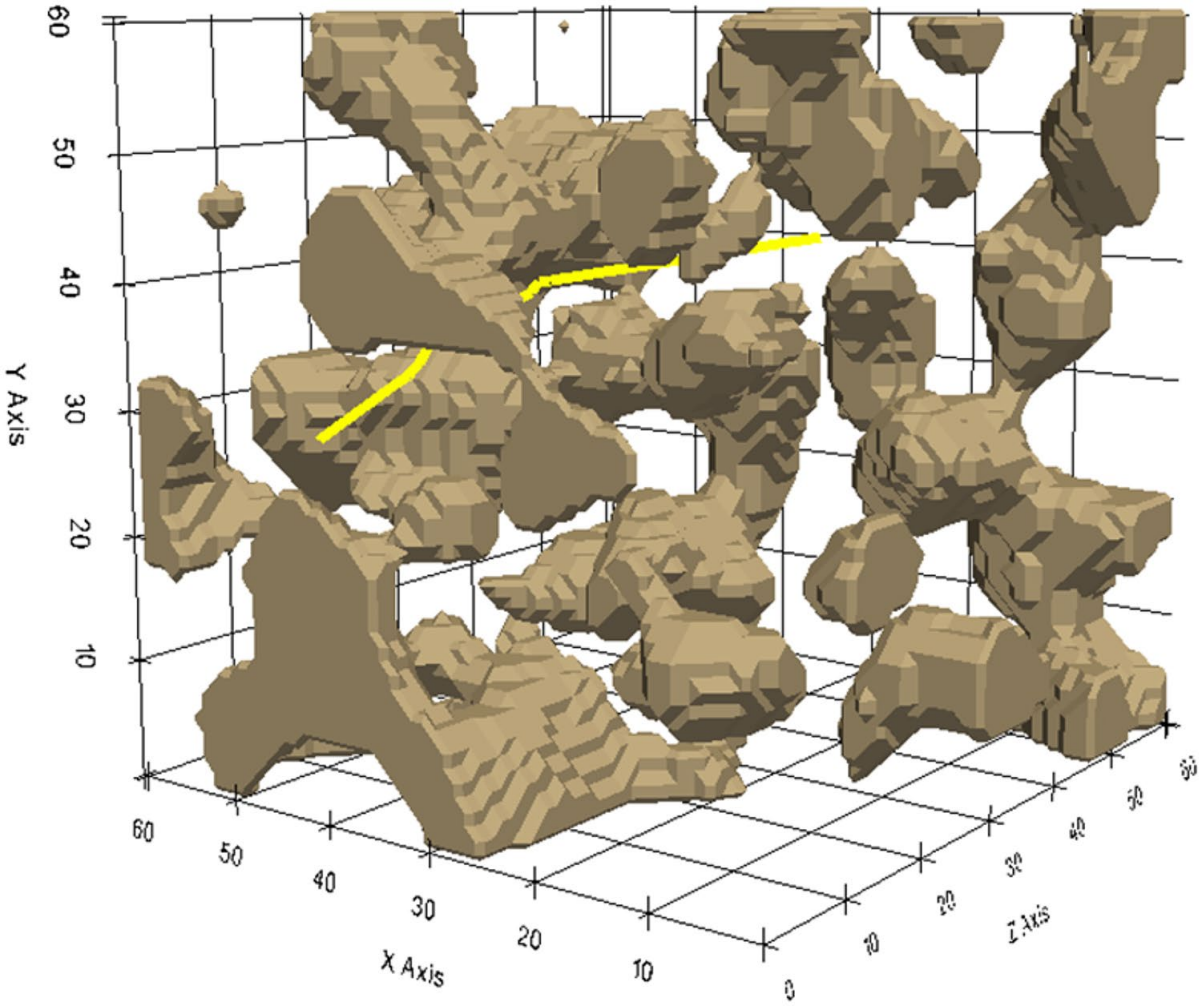
The A-Star algorithm generates the grid map and path in a simplified three-dimensional selected porous medium.

Several paths are generated from the inlet to the outlet surfaces of the porous domain. In Fig. 8, the yellow line represents a possible path generated using the A-star procedure on a 3-D sample. Light brown regions characterize the solid material.

A-Star algorithm finds the shortest route from one node to another, but the number of paths is related to the number of starting and ending nodes. To understand this, call N the number of start nodes and M the number of end nodes, both found considering the Pore Centroid Approach. There will be N times M paths that should be analyzed. Due to the high number of possible paths, only the shortest paths are considered to achieve a maximum of one-hundred paths. Numerically talking, the path with the minimum value of nodes to pass through the medium is selected from the set of paths respective to the starting node. The minimum value of the paths is used on algorithms like the Direct Shortest Path Search method and the Skeleton Shortest Path Search method^5^. Online Appendix D shows a pseudocode of the A-star algorithm.

### Geometric tortuosity

**Figure 9:**
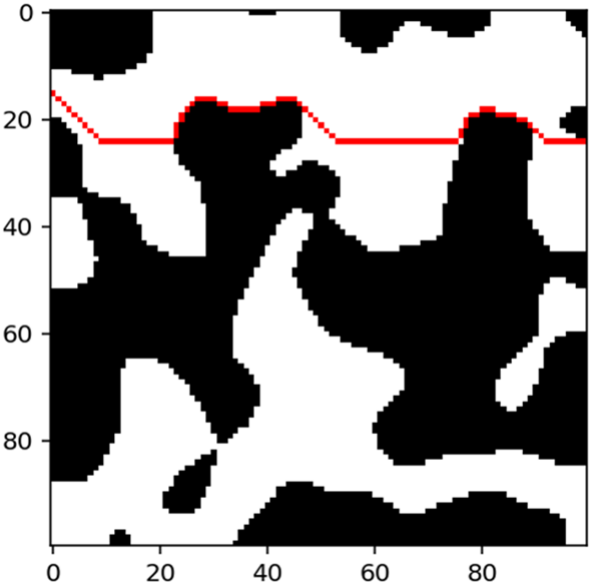
The red line represents a geometric path for tortuosity computation.

To compute the geometric tortuosity, Eq. (1) is applied. It is the ratio between the length of paths and the straight line. Then, the mean value of the paths obtained in the previous procedure is considered as the length of the paths, and the straight line is the length of the domain. Considering the nature of the current study, the geometric tortuosity can be computed as follows:

9 $$\tau =\frac{\frac{1}{m}\sum_{i=1}^{m}[\mathrm{min}({C}_{i})]}{L}$$

where *i* represents the index for the starting nodes, ${C}_{i}$ is the set of paths of the starting node *i*. In addition, *m* is the amount of starting nodes, and L measures the shortest length in the domain, i.e., the side length of the domain, which is equal to 40 in this study. Figure 9 shows a generated path for a 2-D porous media layer.

### Pore size distribution

The PSD is the relative abundance of each pore size in a representative volume of a porous medium. It can be represented by a function *f(r)*, which is proportional to the combined volume of all pores whose effective radius is within an infinitesimal range centered on *r*. The algorithm provided by the Porespy library from Python computes the density function of the pore size distribution by analyzing the histogram of voxel values in the distance calculated over the generated binary matrix that composes the porous media. On the other hand, the cumulative distribution function is also generated, representing the proportion of pore sizes that can fill the pore space.

#### Numerical computation

As mentioned, the PSD function of Porespy is used to describe the pore material. It is executed by the *porespy.metrics.pore_size_distribution* function in Python code^14^. The mentioned function requires the input of four parameters. Given the characteristics of the current study, two of them are established, while the other two are left as default values. The two parameters are *Im* and *Log*, which are defined as follows:

**Im** represents the array containing the sizes of the largest spheres that overlap each voxel. They are obtained from either porosimetry or the local thickness method. These two methods will be explained later in detail.

**Log** is a boolean value; if true, the size data is converted to common logarithm values before processing. In the current study, this parameter is set as false.

#### Methods to extract pore sizes

**Figure 10:**
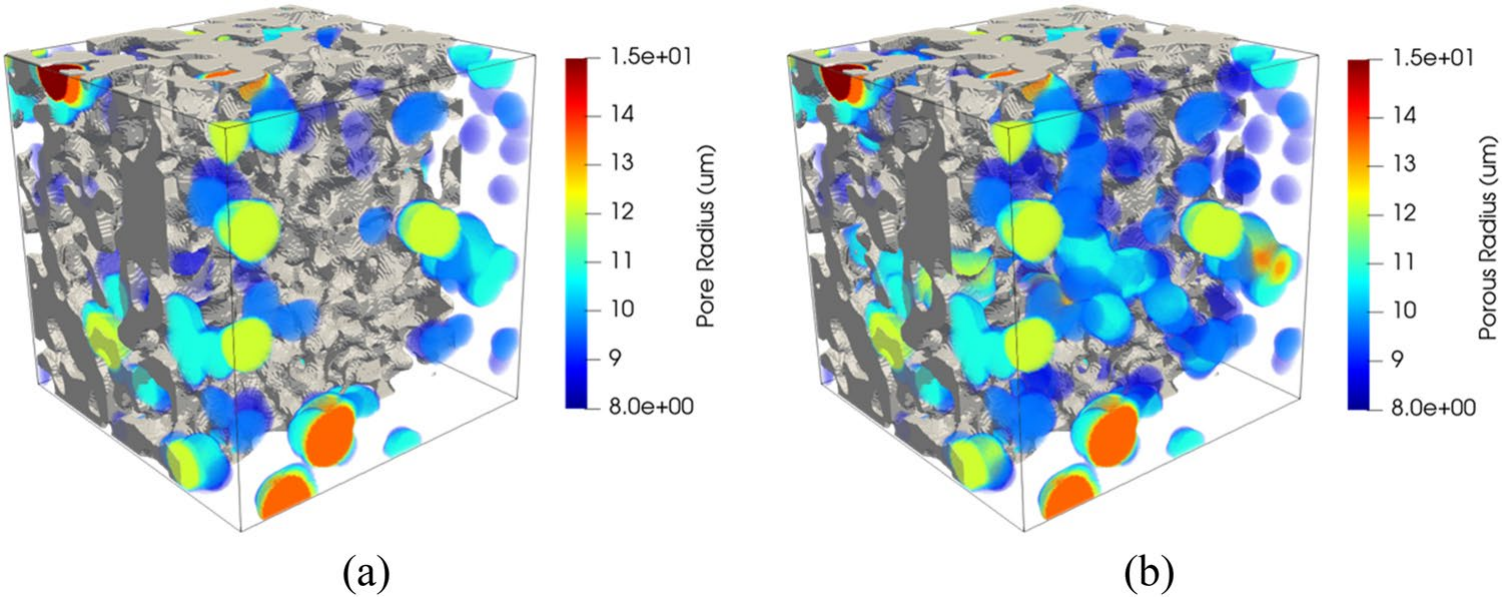
3D-Rendering showing the local pore sizes computed by simulated MIP (**a**), applying the local thickness method (**b**).

The Porespy library has two methods to extract the sizes of pores. These are the porosimetry and the local thickness method^14^. According to Jervis et al.^51^ the main difference between these two methods is that the simulated mercury intrusion porosimetry (MIP) incorporates a shielding effect. Whereby internal pores can be shielded by smaller pores nearer to the exterior of the sample, making them unreachable until higher pressures are applied. The local thickness method gives a more accurate account of the size of all the pores in the model. In contrast, the simulated MIP results provide a pore size distribution closer to the experimental procedure. A porous medium was analyzed with the two methods to show the differences, as presented in Fig. 10. Simulated MIP corresponds to the left image, and Local Thickness results are shown on the right. The colored spheres represent the covered pore spaces for the respective method. To reduce clutter, the pore sizes have been thresholded to the same value (8 µm) for each image. Obtained results show that the simulated MIP method ignores some of the larger internal pores shielded by smaller pores. Since it does not cover larger pores and the media were thresholded, some regions remain empty in that space. While the Local Thickness method does the opposite, more volume is occupied by the larger spheres.

**Figure 11:**
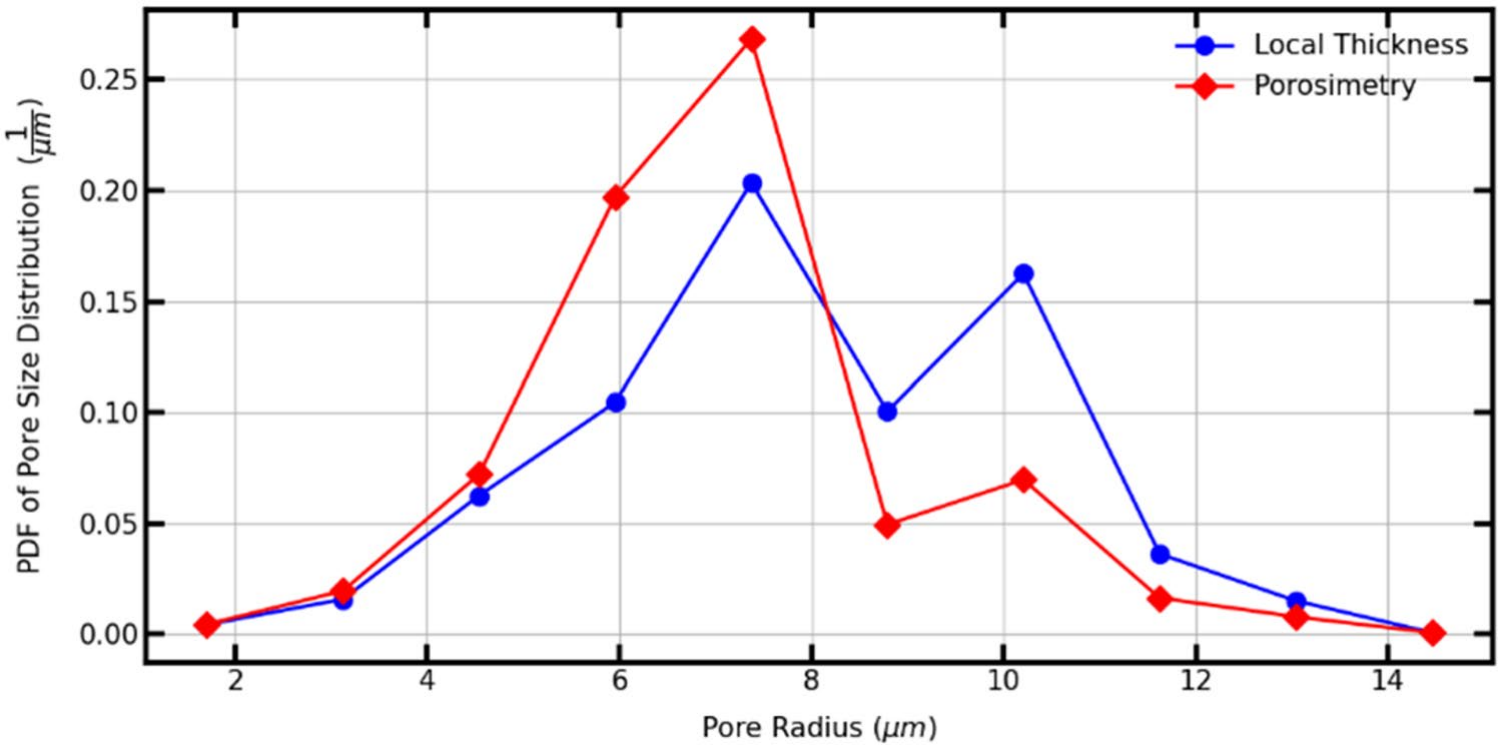
Probability density function (PDF) of pore size distribution for local thickness and porosimetry methods.

It can be corroborated in Fig. 11, where the probability density function (PDF) is computed for both. PDF of Simulated MIP shows that pores of 7 µm have the most significant presence, with the most negligible proportion on greater pore radius. PDF of Local Thickness also indicates the most significant proportion on 7 µm pores, but it has considered greater pore radius since they are not the least values (10 µm) and keeps a slight difference with the greatest. Comparing both, the values obtained by the local thickness method are greater than their counterparts via simulated MIP. This behavior can also be seen in other studies^51,52^. On the other hand, simulated MIP preserves the character of the Gaussian distribution used for the generation of the media since the largest pores are concentrated on the approximate value of 7 µm, unlike the Local Thickness method, where the majority are concentrated on the approximate value of 7 µm and 10 µm. Since this study aims to analyze porous media based on Gaussian generation, the simulated MIP method is preferred to estimate the pore size distribution.

#### Simulated porosimetry function

To implement the simulated porosimetry, the function *porespy.filters.porosimetry* is employed. The input is a *matrix* of the porous media in 2-D or 3-D, setting the void phase as True. The invasion of the void space starts from the surface, giving a shielding effect for the greater internal pores, occupying them with smaller ones. This function computes the pore sizes by performing a distance transform to estimate the largest sphere that could be centered on each voxel^14^, and applying a second distance transform to obtain the invading fluid configuration in the trimmed mask. As a result, a copy of the initial porous media with the pore size values in each voxel is generated as a matrix.

## Results and discussions

### Generated porous media

Table 1 presents examples of the 3D artificial porous media generated by Python and rendered by Paraview. As mentioned, 3300 samples were generated, fifty for every different porosity and blobiness value in the range of 0.45–0.95 and 0.50–1.00, respectively. Porous media with the minimum and maximum values for porosity and blobiness are shown to observe the impact of the mentioned variables. The porous media generated tends to have fewer particles while porosity increases. Also, these particles become smaller and spread more when the blobiness increases.

**Table 1:** 3-D Rendering of artificial porous media for this study.

φ\δ	0.5	1.0
0.45		
0.95		
	Porosity = φ	Blobiness = δ

Minimum and maximum values of porosity and blobiness are considered.

Since the sigma value in the current research is a function of the blobiness value, it can be evaluated on Eq. (4). Table 2 presents the sigma value obtained for the current study parameters.

**Table 2:** Sigma function evaluation considering the blobiness range from 0.5 to 1.0 and sample size 40.

Blobiness ($\delta )$	Sigma $\upsigma (\delta )$
0.5	2.00
0.6	1.67
0.7	1.43
0.8	1.25
0.9	1.11
1.0	1.00

As shown in Table 2, the sigma value falls in the range of 1.0–2.0 while the blobiness decreases. For the Gaussian filter, as the sigma value gets high, it will increase the number of calculations per pixel, making the image blurred. To relate the current study directly with the Gaussian filter, for the following sections, the sigma value will be reported in place of the blobiness value.

### Tortuosity-porosity-pore size correlations

#### Tortuosity as a function of porosity

In Table 3, tortuosity correlation as a function of porosity for each blobiness value is presented. Each correlation is computed based on 550 samples on a porosity range of 0.45–0.95 with steps of 0.05. The R-square and Sum of squared error (SSE) of interpolated function, statistical approaches used to determine the goodness of fit on interpolation or linear regression, guarantee the correlation’s accuracy. The best correlations were selected based on the mentioned statistical parameters.

**Table 3:** Empirical tortuosity–porosity relations for Porous media.

Sigma	Correlation for tortuosity	SSE (× 10^−3^)	R-square
1.0	$2.111*{\mathrm{e}}^{-0.7286\mathrm{\varphi }} + 3765*{\mathrm{e}}^{-24.07\mathrm{\varphi }}$	3.566	0.9901
1.11	-18.72 $*{\mathrm{\varphi }}^{0.033 }+19.74$	3.050	0.9887
1.25	$15.12*{\mathrm{e}}^{-12.15\mathrm{\varphi }} + 1.835*{\mathrm{e}}^{-0.5993\mathrm{\varphi }}$	2.236	0.9904
1.43	$1.811*{\mathrm{\varphi }}^{-0.2643 }-0.8012$	2.720	0.9871
1.67	$7.695*{\mathrm{e}}^{-9.355\mathrm{\varphi }} + 1.634*{\mathrm{e}}^{-0.4885\mathrm{\varphi }}$	3.177	0.9839
2.0	$5.98*{\mathrm{e}}^{-7.666\mathrm{\varphi }} + 1.44*{\mathrm{e}}^{-0.3665\mathrm{\varphi }}$	5.983	0.9682

**Figure 12:**
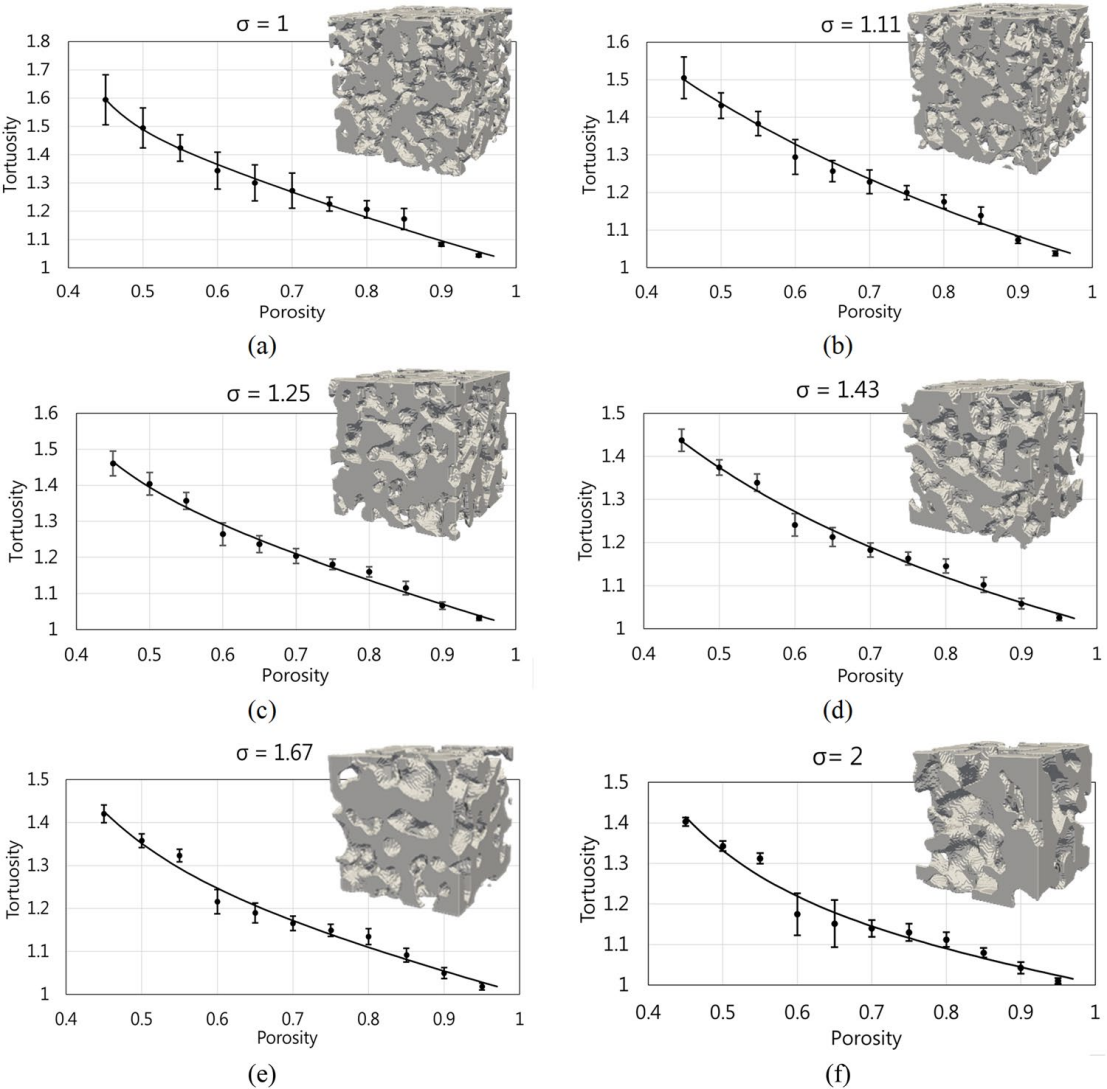
The continuous black line corresponds to the graph representation of correlations from Table 3. For each geometric tortuosity value, their corresponding standard deviation is presented. 3D rendering samples are presented to analyze the impact of the sigma value. All the selected samples correspond to the porosity of 0.5, and the sigma value increases according to its blobiness value.

Correlations from Table 3 are depicted in Fig. 12 considering the error bars as result of the standard deviation of the computed data.

As the graphs in Fig. 12 show, the tortuosity behavior changes slightly as the pore size increases. The most noticeable changes that allow them to be differentiated are observed when the porosity has a value of 0.45 since it can be seen that as the pore size increases, the tortuosity decreases. The highest value of tortuosity is approximately 1.6, which occurs when the pore size is small in Fig. 12a. And the approximate tortuosity value of 1.4, occurs when the pore size is larger in Fig. 12f. This reduction in tortuosity is appropriate since the media generation algorithm, by increasing the sigma value, generates a more compact grain distribution, which causes pore connectivity within the media to improve and larger pores are allowed form. From Fig. 12 it can be seen that by having larger pores, the complexity or resistance to the passage of species within the porous medium is reduced. In addition, when the porosity is the highest, in this case, 0.95, all media approximate their tortuosity to 1, as known theoretically. The behavior of the geometric tortuosity at intermediate porosity values, i.e., ​​ranging from 0.50 to 0.90, although the data have been fitted with a potential function, it is noticeable that the intermediate points of tortuosity do not completely follow this relationship, and this variation or error between the measured value and the adjusted value increases as the pore size increases. Starting with Fig. 12a, it can be seen that at porosities from 0.60 to 0.70 adjusted tortuosity values are obtained that slightly overestimate the real values. On the other hand, porosity values from 0.80 to 0.85 show tortuosity values below the real values. This relationship holds in Fig. 12b. Increasing the pore size, as shown in Fig. 12c, for the porosity of 0.55 the tortuosity begins to generate an error by underestimating the real value. Presenting this error with a slight increase in Fig. 12d as well as in the tortuosities of porosities from 0.60 to 0.65 and from 0.80 to 0.85 in the same figure. This error and especially the difference between the tortuosity values between 0.55 and 0.60 becomes quite noticeable in Fig. 12e,f. This means that the resistance between such porosities changes abruptly. On the other hand, the connectivity between them could have played an important role. An important factor to consider in this situation is the randomness of the media. This event can be a subject of study for future work.

#### Comparison with two-dimensional random porous media

To discuss the implications of three-dimensional tortuosity computation, a recently done tortuosity study in 2D porous media is compared. It shows clear differences in both the methodology and the computational power required. First of all, despite being the same A-star algorithm, the method of obtaining paths consumes less computational power in two-dimensional porous media. This is due to the number of nodes that this algorithm must evaluate. While for a two-dimensional medium, considering samples of square sizes $n$ the nodes can be ${n}^{2}$, for a three-dimensional considering sample of cubic sizes, they can be ${n}^{3}$. And this process is repeated for each input node ${n}_{in}$. In other words, for a two-dimensional medium, ${n}_{in2D}$*${n}^{2}$ nodes must be analyzed, while for a three-dimensional medium, ${n}_{in3D}$*${n}^{3}$ nodes must be analyzed for path estimation. And depending on the resolution of the images, n can take values of 50, 100, 500 or larger and can reach values of hundreds of millions or even billions of nodes, as well as the computational resources allow. As mentioned by Fu et al.^5^, for 3D porous media characterization it is common to use image processing techniques that allow reducing the dimensions of porous media at the cost of losing geometric characteristics.

On the other hand, for 3D computation estimating a representative node for each layer or 2D slice using techniques such as pore centroid is considered a suitable methodology when performing downsizing. Although a factor to take into account with this methodology is that it must be guaranteed that each layer has a pore that allows connectivity with the previous pore, if this is not fulfilled, an adequate geometric tortuosity cannot be estimated. As mentioned before, the pore centroid technique was used to estimate the starting and arrival pores of the paths to be generated to guarantee the calculation of paths that our computational resources allow. Reducing the number of nodes by reducing the number of input nodes ${n}_{in}$.

By comparing with numerical data, the selected paper done by the co-authors of this study is presented^43^. This article worked with two-dimensional artificial porous media. The porosity that this study evaluated was in the range of 0.50–0.90. The sigma used was 2. Therefore, to perform an adequate comparison, the correlation obtained in Fig. 12f is selected and compared with the one obtained in the study of 2D porous media.

**Figure 13:**
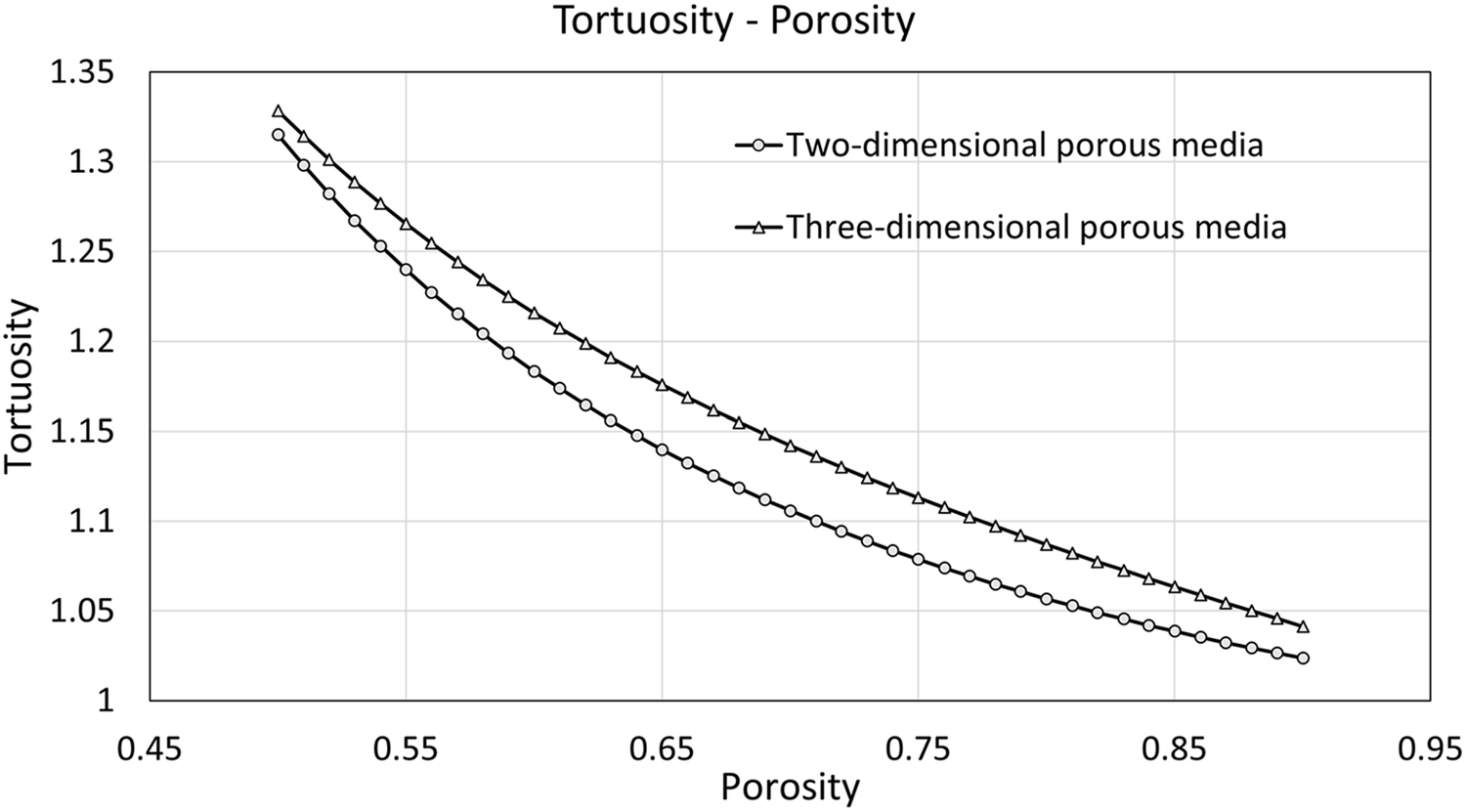
Comparison between tortuosity-porosity correlations from a two-dimensional study and the current three-dimensional study.

Figure 13 presents a graphical comparison between the correlations for the pore structures presented in Table 4.

**Table 4:** Empirical tortuosity–porosity relations for porous media at two and three-dimensional pore structures.

Pore structure	Correlation for tortuosity
Two-dimensional random porous media	$9.112*{10}^{-2}*{\varphi }^{-2.155}+0.9093$
Three-dimensional random porous media	$5.98*{\mathrm{e}}^{-7.666\mathrm{\varphi }} + 1.44*{\mathrm{e}}^{-0.3665\mathrm{\varphi }}$

According to Fig. 13, the present study denotes that three-dimensional porous media have greater tortuosity than two-dimensional porous media in the range of 0.50 to 0.90. This can be explained due to the nature of the geometric tortuosity calculation. Although in both cases the A-star algorithm has been used, the paths in the three-dimensional porous medium will be longer than in the two-dimensional media, which will contribute to the increase in the distance traveled within the medium. Following the definition of tortuosity in Eq. (1), increasing the distance traveled will increase the geometric tortuosity. Of course, it must be considered that according to Fig. 13 both correlations do not have such a distant difference in values in geometric tortuosity and this may be due to the difference in size in the media studied. While in the two-dimensional study it was treated with media of 100 × 100 pixels, in the present study it was treated with media of 40 × 40 × 40 voxels. The difference is that in the 2D case, the possible nodes to analyze are ${n}_{in2D}*{10}^{4}$ and in the 3D case, they are ${n}_{in3D}*64*{10}^{3}$. Despite being distant values, the number of inputs can adjust so that the nodes to analyze are closer. And keep in mind that the maximum input limit for both studies is 100, for 3D due to the porous media cutoff algorithm while in the 2D study due to the size of the medium. The possibility that the 3D algorithm has fewer inputs is greater since when cutting the porous medium, many input nodes become one. In case of not obtaining connectivity with the next node, the other possibilities that were not the centroid of the cut but that possibly had an effective path are lost.

On the other hand, something different can happen outside the established range, since it can be assumed that at values less than 0.45 the tortuosity of the porous medium in two dimensions will become greater than that obtained by the porous medium in three dimensions. This is very likely since even though there may be more nodes to analyze in the 3D porous medium than in the 2D one. As the porosity decreases, the 2D porous medium begins to run out of paths faster, so the resistance of the porous medium to the passage of species increases more. This occurs because the 2D porous medium does not have a transverse axis as occurs in the 3D porous medium, where in case there is no adjacent node in the direction of flow, the transverse nodes can be searched and an effective path can be found.

#### Comparison with previous studies on different porous structures

This section shows a comparison between the obtained correlations and theoretical and empirical correlations found in the literature. Table 5 contains the correlations with their corresponding porous structure characteristic. Previous correlations were taken from different authors representing 3D porous media tortuosity^53–55^.

**Table 5:** Theoretical and empirical tortuosity–porosity relations for porous media obtained from previous studies for different pore structures.

Pore structure	Correlation for tortuosity	Author
A hyperbola of revolution	$2-\varphi$	Petersen (1958) and Rayleigh (1892)^53^
Cubic particles	$1-\frac{\varphi }{2}+\frac{{\left(1-\varphi \right)}^\frac{1}{2}}{4}+\frac{\left(\varphi +1+{\left(1-\varphi \right)}^\frac{1}{2}\right)*{\left(9-5\varphi -8{\left(1-\varphi \right)}^\frac{1}{2}\right)}^\frac{1}{2}}{8\varphi }$	M. Yun et al. (2006)^55^
Random packs of grains	${\varphi }^{-\frac{1}{2}}$	Bruggeman (1935)^53^
Overlapping spheres	$1-\frac{\mathrm{ln}\left( \varphi \right)}{2}$	Akani et al. (1987)^53^
Granular beds	${\varphi }^{-0.3655}$	Archie (1942)^54^
Partly saturated homogeneous isotropic monodisperse sphere packings	${\varphi }^{-\frac{1}{3}}$	Millington et al. (1959)^53^

**Figure 14:**
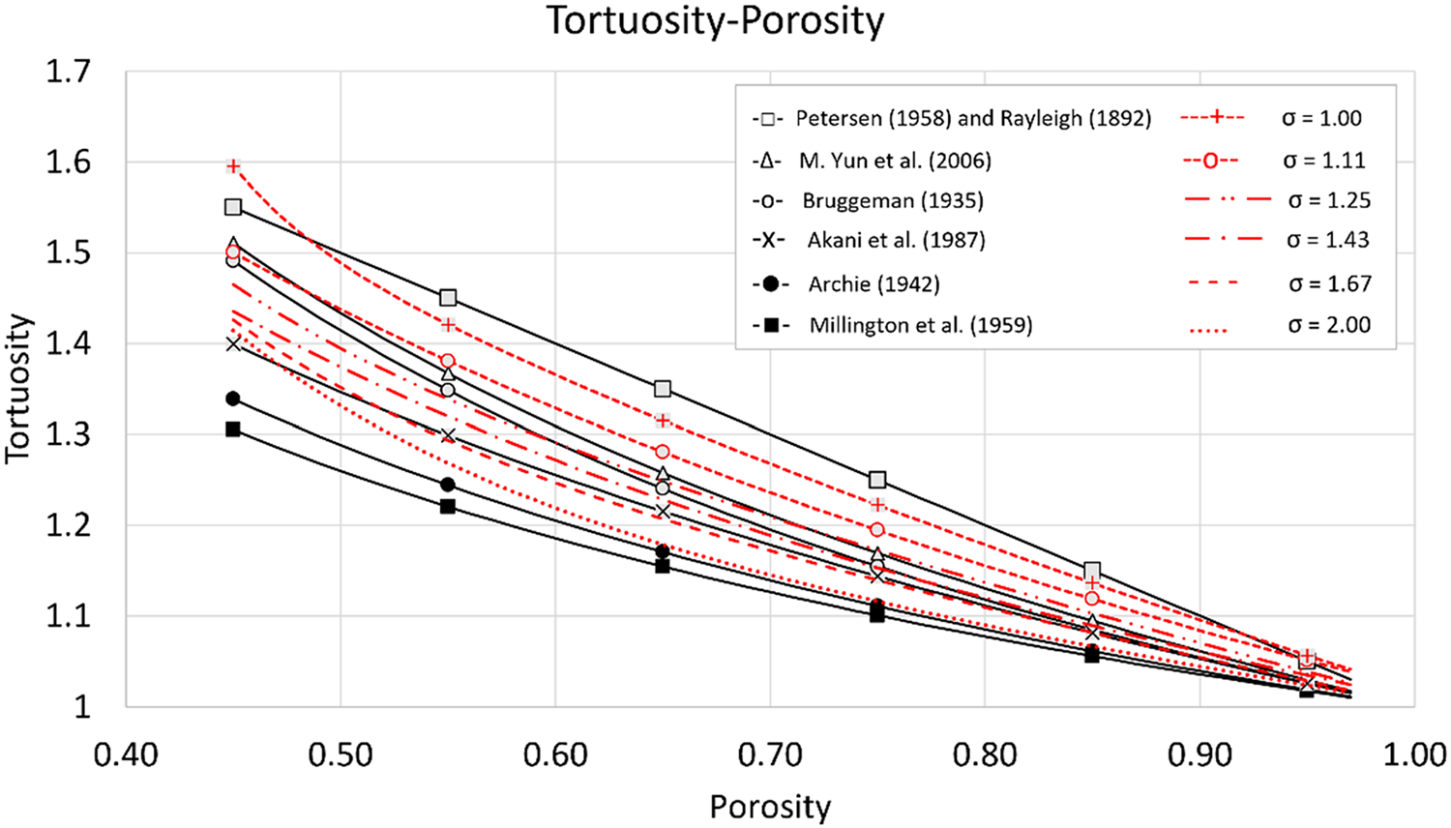
Comparison between tortuosity-porosity correlations from previous studies (black markers and lines) and correlations proposed in the current study (red markers and lines).

Figure 14 presents a graphical comparison between the correlations for different pore structures from previous works summarized in Table 5 and the proposed correlations presented in Table 3.

As shown in Fig. 14, the correlations proposed in the current study fall in the region of the previously demonstrated correlations. At first, all the proposed correlations of this study overpredict the proposed by Archie and Millington. This result can be associated with the porous structure of both authors, which usually preserves a homogeneous particle distribution and contributes to that the fluid invasion was not restricted by complex pore spaces, achieving lower tortuosity values. On the other hand, for the porosity range of 0.50 to 0.85, the correlation of Akani et al. overlaps the curves of porous media generated with sigma values of 2.0 and 1.67. This result would imply that the proposed model of overlapping spheres has more complex pore spaces and more significant tortuosity. At the same time, the Bruggeman equation for the porosity range of 0.45–0.75 overlaps the curves of sigma lower than 1.43. The same behavior is repeated by Yun et al. curve but in the porosity range of 0.45–0.85 with a higher tortuosity. For the range of 0.50–0.95, the sigma 1.0 and 1.11 curves overlap the already mentioned correlations with a higher tortuosity. Finally, for the range of porosity of 0.50–0.95, the correlation proposed by Petersen and Rayleigh overlays all the proposed curves in the current study. As expected, for all the curves, the lower the porosity, the higher the tortuosity is. At the same time, when the porosity tends to the unity, the tortuosity also does. In addition, lower sigma results in greater geometric tortuosity values. This means the paths become more obstructed and narrower as the sigma decreases.

#### Tortuosity as a function of porosity and sigma

**Figure 15:**
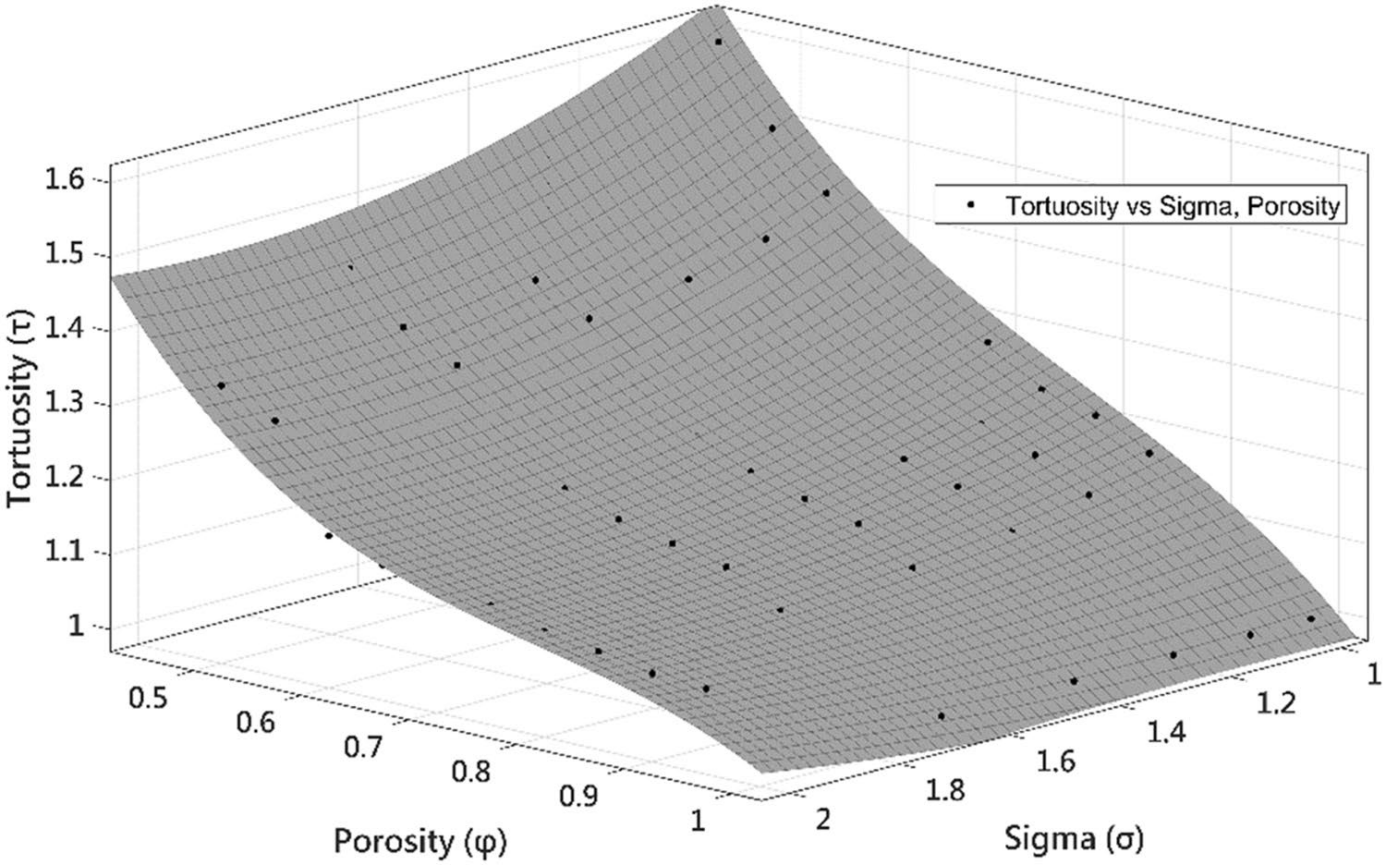
Surface behavior representing the tortuosity values as a function of the porosity and sigma. The surface is generated from the equation in Table 6.

As mentioned, studies have demonstrated that tortuosity depends not only on the porosity, but another morphological characteristic has to be considered because it directly affects the paths of the media. Table 6 shows a tortuosity correlation as a function of porosity and sigma since its last parameter controls the distribution of particles on the generated porous media. Figure 15 shows the 3D multivariable function considering the simulated results and fitting tools.

**Table 6:** Empirical tortuosity–porosity-sigma correlation for porous media.

$$\tau \left(\sigma ,\varphi \right)=4.059 -0.924*\sigma -7.173*\varphi + 0.2995*{\sigma }^{2}+0.1716*\sigma *\varphi +7.91*{\varphi }^{2}-0.2329*{\sigma }^{2}*\varphi +0.5834*\sigma *{\varphi }^{2}-3.702*{\varphi }^{3}$$	
Type	Polynomial
SSE	0.02101
R-square	0.986

Sum of squared error of prediction (SSE) and R-squared for the possible correlation.

According to the statistical parameters, the proposed surface equation can predict 98.6% of the tortuosity values as a function of the porosity and sigma with a 95% confidence interval.

### Pore size distribution

Pore size distribution (PSD) is computed and represented by the cumulative and probability density functions. The influence of blobiness on the PSD is analyzed by a graph comparison of different samples. For explanation purposes and considering the minor number of samples to use, there were considered porous media with larger sizes than the analyzed in the data collection process. Three-dimensional porous media of 100 × 100 × 100 voxels with 0.5 porosity were used.

#### 3D simulated porosimetry pore sizes

**Figure 16:**
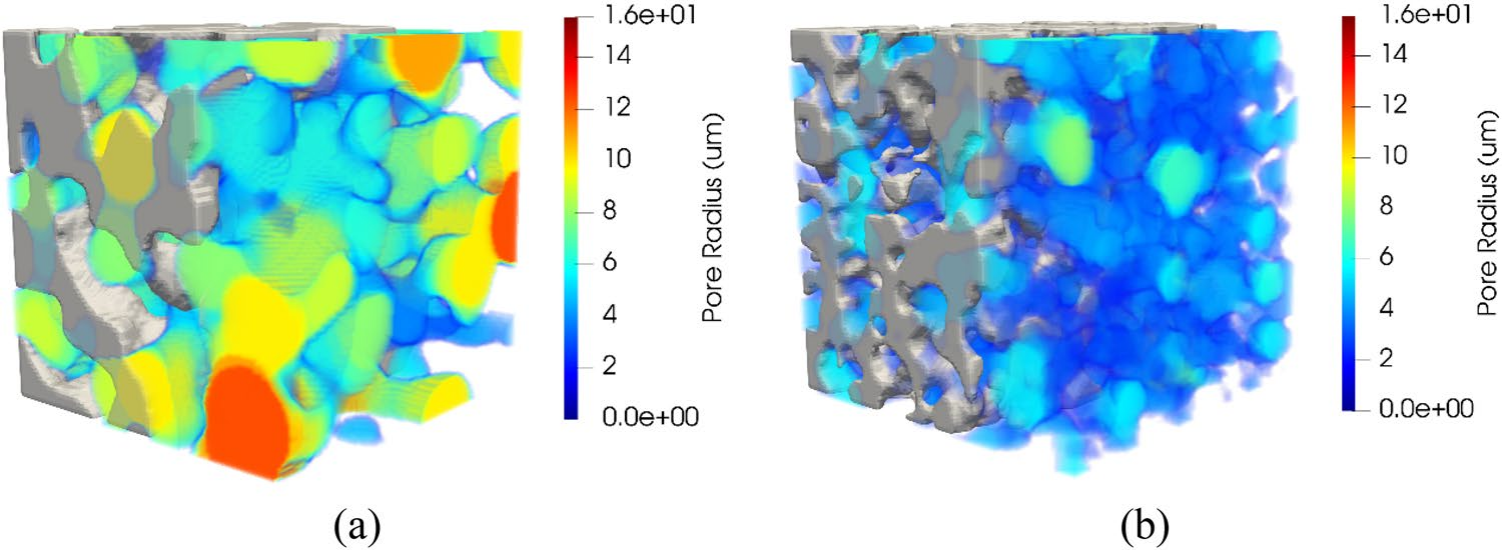
Rendering of selected samples showing the local pore sizes computed by simulated porosimetry method for porous media with porosity of 0.5 and sigma of 2.0 (**a**) and 1.0 (**b**).

Two porous media samples were generated and presented in Fig. 16. For both samples, the porosity is established at 0.5, and the sigma value is 2.0 (left) and 1.0 (right). Simulated porosimetry methods were applied to obtain the pore size distribution of the samples. The 3D rendering allows visualizing the pore sizes inside the material domain. As a result, larger pore sizes are frequent when porous media with sigma equal to 2.0 are generated. On the other hand, smaller pore sizes are more common when sigma is 1.0. It can be observed in the vast presence of green, yellow, and red spheres in the case of sigma 2.0 and blue spheres in sigma 1.0.

#### Probability density function

**Figure 17:**
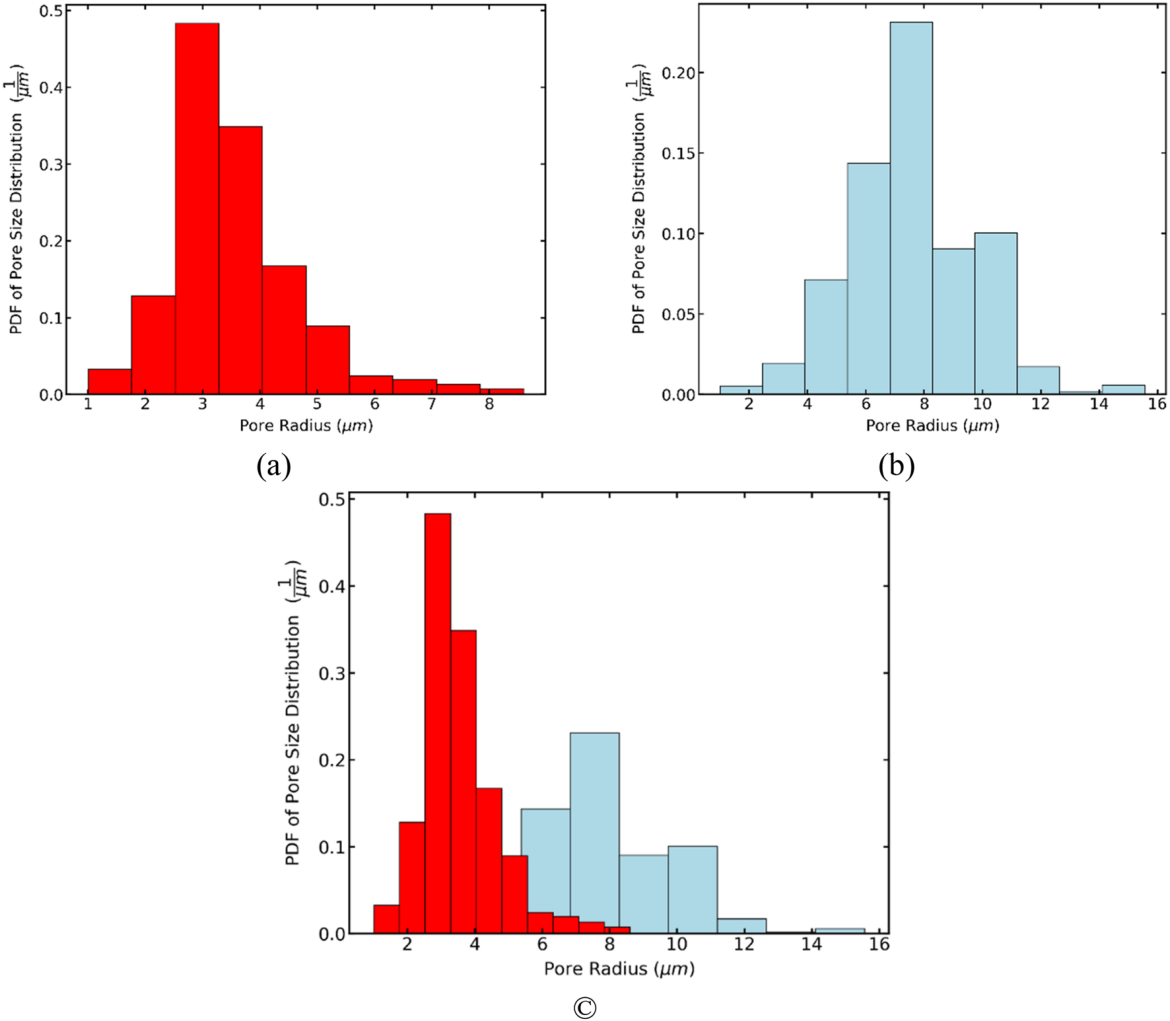
Histograms of probability density function (PDF) of the pore size distribution (PSD) for porous media with porosity of 0.5 and sigma of 2.0 (**a**) and 1.0 (**b**). A comparison between the two different sigma values (**c**).

To determine the probability density functions, two different media are generated. Histograms of PDF are presented in Fig. 17. A porous medium with sigma 2.0 is illustrated in Fig. 17a, while Fig. 16b shows a sample with sigma 1.0. A comparison is shown in Fig. 17c. Porous media with a sigma of 1.0 has smaller pore sizes, contrary to the porous media with sigma 2.0, which has bigger pore sizes. Another interesting thing is that both follow a Gaussian distribution. This can be explained by how the samples are generated, i.e., the effects of the Gaussian Blur.

**Figure 18:**
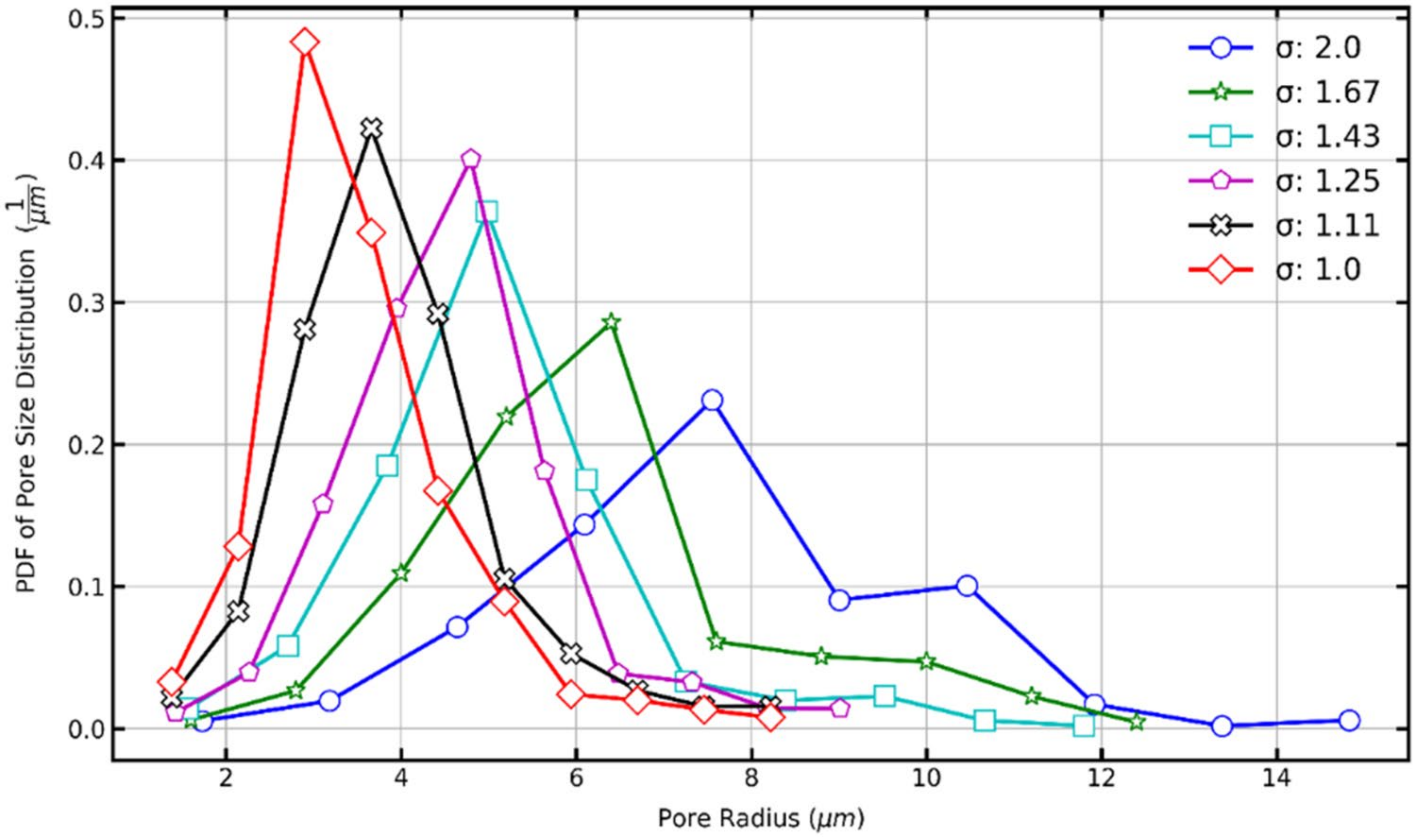
Probability density function (PDF) of pore size distribution of porous media with sigma 1.0–2.0

Figure 18 shows the PDFs generated for the sigma values used in this study. As a result, it could be stated that as the sigma value decreases, the probability of finding porous media with pores of smaller radius increases. On the other hand, the increment of the sigma value implies a presence of larger pore sizes but in smaller amounts.

#### Effect of average pore size on tortuosity

For the porous media that formed the pore size distribution in Fig. 18, the geometric tortuosity and the average pore size were estimated. Due to the size of the porous media, which were 100 × 100 × 100 for this analysis, the A-star algorithm was not adequate to estimate the geometric tortuosity since the computer’s RAM memory filled up after a while without having estimated a path. Therefore, for this section, the tortuosity calculation is purely performed with the Pore Centroid method. This indicates that to estimate the geometric tortuosity it is enough to estimate the value of the Euclidean distance formed by the centroids present in each of the 2D layers that form the 3D porous medium.

On the other hand, the average pore size was estimated by fitting the data of the probability density function presented in Fig. 18 to a Gaussian density function using a curve fitting tool. Therefore, the average pore size was estimated for every sigma value and presented in Table 7.

**Table 7:** Average pore size evaluation for every sigma’s pore size distribution.

Sigma $\upsigma$	Average pore size ($\mathrm{\mu m})$
1.00	3.371
1.11	3.700
1.25	4.510
1.43	4.942
1.67	5.839
2.00	7.416

**Figure 19:**
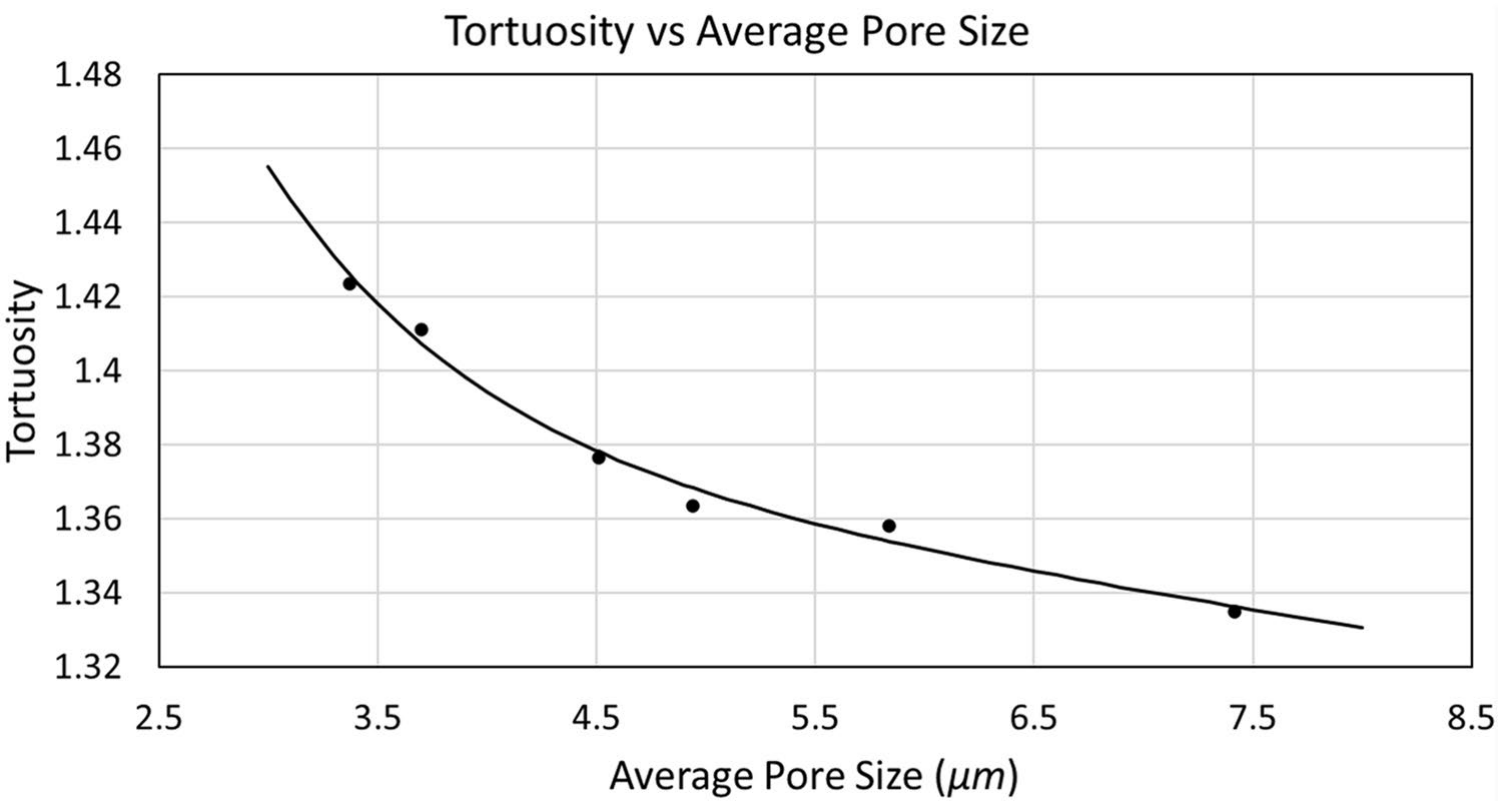
The continuous black line corresponds to the fitting function of the tortuosity depending on the average pore size for the porous media.

As Table 7 shows, the average pore size is greater if the sigma value increases. The mentioned occurs since it controls the particle arrangement, therefore when the particles have a compact form (sigma increases), the conductivity of the porous media tends to be represented by greater pore sizes. After estimating the geometric tortuosity and the average pore size, an exponential correlation was obtained:

Figure 19 shows a representative graph of correlation presented in Table 8.

**Table 8:** Empirical tortuosity–average pore size correlation for porous media.

Correlation for tortuosity	SSE (× 10^−5^)	R-square
$1.921*{e}^{-1.065*\rho } + 1.405 *{e}^{-0.006834*\rho }$	6.635	0.9882

Sum of squared error of prediction (SSE) and R-squared for the possible correlation. Where $\rho$ is the average pore size.

As shown in Fig. 19, the tortuosity decreases as the average pore size increases, as previously mentioned in this study. The resistance of the medium to the passage of species becomes smaller when increasing the space of the routes by which flowing. To make a comparison with the data presented in Fig. 12, the differences between both generated media must first be taken into account. In the first place, for the media obtained in Fig. 12, sizes of 40 × 40 × 40 were considered using the A-star methodology. While for the media in this section, sizes of 100 × 100 × 100 were obtained using the Pore Centroid methodology. In this section at a porosity of 0.50 and average pore size of 7.416 µm, a tortuosity of 1.3347 was estimated, while in Fig. 12f, at the same porosity and its corresponding sigma of 2, the estimated tortuosity was 1.3348 using the correlation presented in Table 6. Despite the case study is random, it can be seen that the calculated tortuosity is quite similar between both, being different sizes. However, this tortuosity begins to vary as the pore size decreases. For a porous medium with an average pore size of 5.839 µm, a tortuosity of 1.358 is obtained, which differs from that presented in Fig. 12e since, at a sigma of 1.67 and a porosity of 0.50, the tortuosity is 1.345. This is a strange case since at a smaller size, the tortuosity should be greater than the larger medium. It can be said that in this case, the smaller medium had better connectivity between the pores than the larger medium. The data begin to make sense since there is an average pore size of 4.942, which gives a geometric tortuosity of 1.363, while in the smaller medium of Fig. 12d, the geometric tortuosity at a porosity of 0.50 is 1.372, which exceeds the largest medium. This relationship is maintained until reaching the smallest of the average pore sizes, corresponding to 3.370 µm. Where the tortuosity for the larger medium is 1.423, while the tortuosity for the smaller medium presented in Fig. 12a, at a porosity of 0.50, is 1.478. This analysis corroborates the mentioned in previous studies^5^ where it is said that the size of voxels of the porous medium is reduced. The tortuosity tends to increase because the space through which species can pass is reduced, even if the same medium is maintained, the geometric information can be lost as the size of voxels decreases. Furthermore, this becomes more noticeable when the pore size is altered. Since even if the pore size is reduced in similar proportions, the tortuosity will increase more when the medium has a lower dimension in voxels.

It is worth mentioning that the A-star method can give a better idea of the complexity of the medium because it considers more paths than the Pore Centroid method which only considers one. The A-star algorithm becomes inefficient as the size of the medium increases. Of course, the pore centroid method is a good alternative for media of considerable size, as in the case seen in this subsection. Even, as mentioned by Fu et al.^5^, the geometric tortuosity estimated with the pore centroid method can be preserved even when the resolution of the same medium is varied since it tends to consider the same path. This is an issue that can be evaluated in randomized means in future studies.

#### Cumulative distribution function

**Figure 20:**
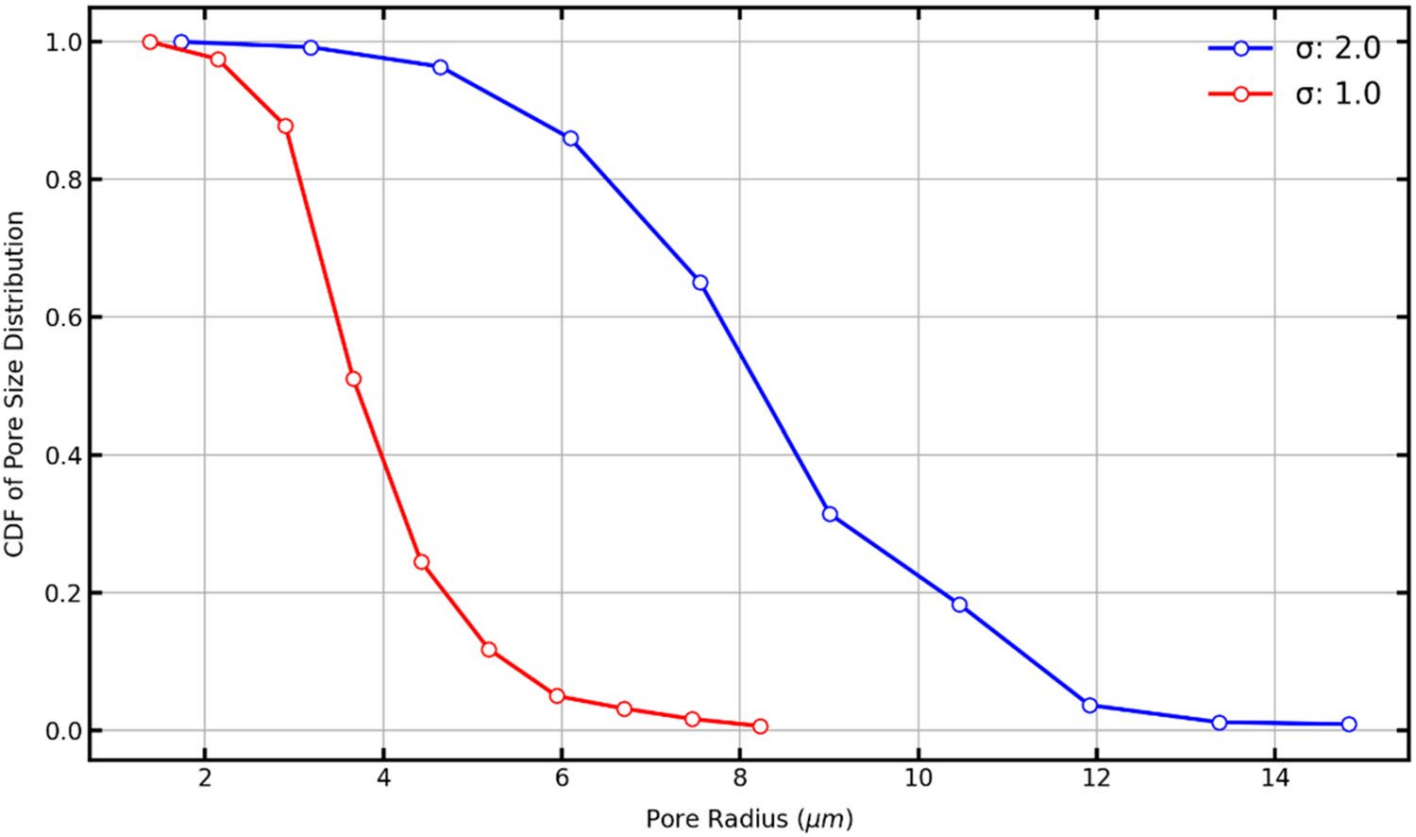
The cumulative density function of the pore size distribution of porous media with a sigma of 2.0 (blue line) and 1.0 (red line).

The cumulative distribution function (CDF) data are shown in Fig. 20 for sigma 2.0 in blue and sigma 1.0 in red. It works as a pore volume fraction graph, indicating that the respective pore radius can fill a fraction of the pore spaces. It is observed that when sigma is 1.0, almost 40% of the pore space can be filled by spheres bigger than 4 µm. Also, 80% of the pore space can be filled by spheres of a radius of almost 3 µm. On sigma 2.0, nearly 30% of the pore space can be filled by spheres of a radius of nearly 9 µm. Also, the 10% can be filled by spheres slightly larger than 11 µm.

**Figure 21:**
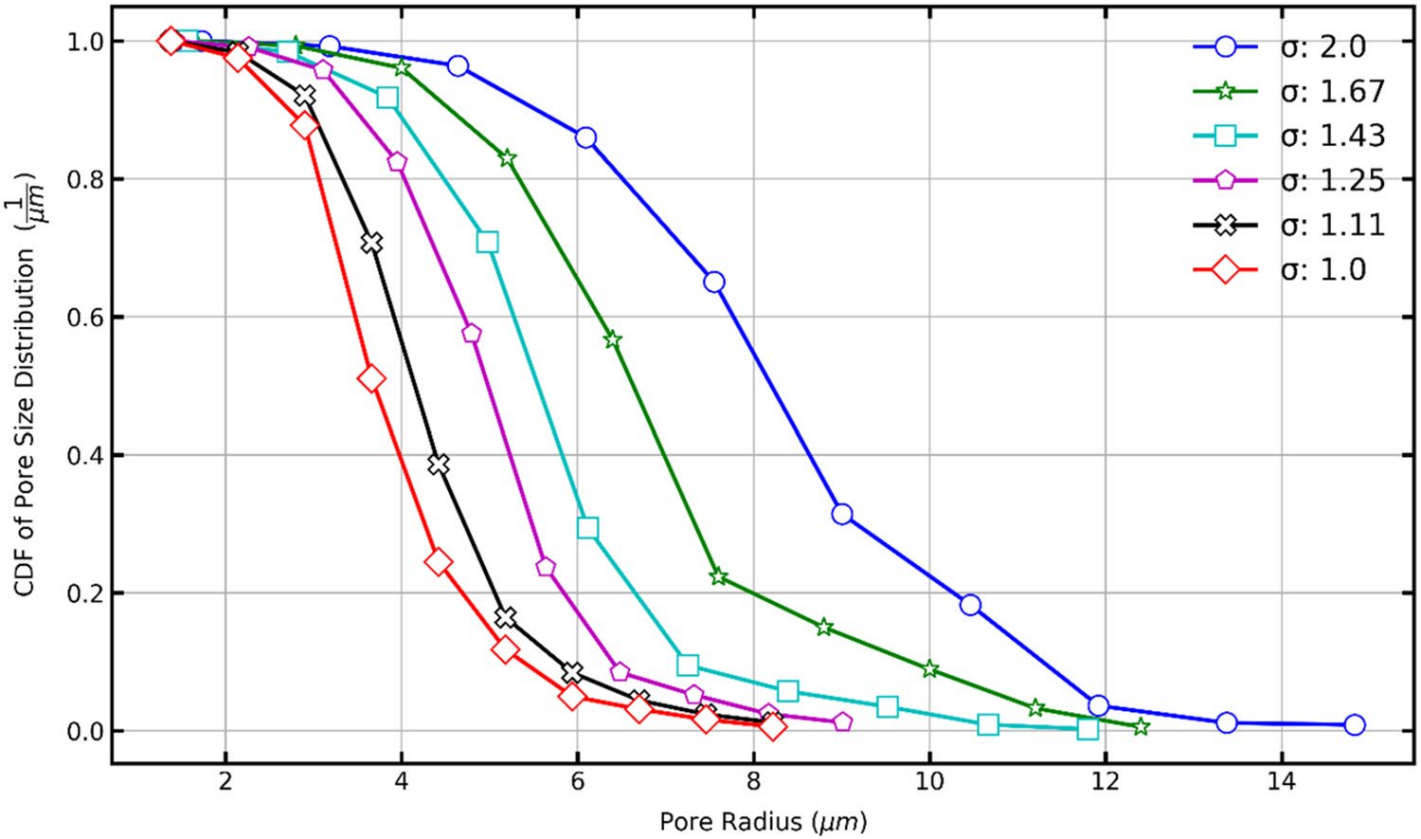
The cumulative density function of the pore size distribution of porous media with sigma in the range 1.0–2.0.

Figure 21 shows a complete comparison with the sigma values of the current work. The tendency again can be seen that pore spaces become bigger when the sigma decreases. Greater spheres can fill a more significant pore space fraction.

## Conclusions

The implications of this study on the characterization of porous media are several. In the first instance, the effect of pore size on tortuosity could be corroborated. When comparing different media with the same porosity, the geometric tortuosity varied considerably as the pore size distribution changed. It can be established that porous media increase their capacity to allow the passage of species as the size of the pores increases. This implies that correlations of tortuosity with porosity alone must be neglected. Rather, the geometric tortuosity must begin to relate to other microstructural variables to achieve a better representation of reality. On the other hand, this tortuosity variation effect does not depend only on the pore size but also on the size of the medium. Since the approximate values of geometric tortuosity were observed to vary, they increased as the size of the medium was reduced. And this is somewhat feasible because as the medium shrinks, the number of nodes to analyze also shrinks, resulting in fewer possible paths to allow species to pass through. It should be noted that in our study, the sigma parameter can be used indistinctly from the size of the medium to relate a pore size distribution since this value alters the grouping of the grains within the medium.

There is also the option of evaluating the average pore size, however, due to randomness, estimating this parameter directly from the generation parameters such as sigma will require taking a lot of data since it will depend on the size of the medium and it is not something that can be estimated. With complete certainty since there is still no correlation with the size of the medium or any other characteristic factor. Therefore, the adjustment technique to a Gaussian function for each porous medium was the best way to obtain the average pore size.

The size domain is also another important factor that can alter the geometric tortuosity. As observed in this study, when comparing means of sizes 40 × 40 × 40 voxels with means of 100 × 100 × 100 voxels. It was observed that as the pore size increased, the tortuosity variation was greater in the smaller medium. This is due to the restriction of the paths being greater in the medium with fewer voxels, since it had fewer nodes through which to form a path, the passage of species within the medium would be more affected.

The algorithm to estimate the geometric tortuosity plays an important role. For this study, the A-star algorithm was mostly used due to its characteristic of evaluating most of the porous medium. This covers larger nodes than other methods specifically designed to obtain a representative path such as the pore centroid method. It can be established that the use of the algorithm depends on the size of the porous medium to be analyzed, that is, the resolution of the medium in voxels. This is because the A-star algorithm analyzes more nodes, and requires greater use of computational resources, which implies that to perform calculations such as those shown in this study, good performance computers are required. Unlike the pore centroid method which estimates a single path based on the pore centroids of each 2D layer, saving computational resources. In this study it was possible to obtain adequate geometric tortuosity values for media of 100 × 100 × 100 using the pore centroid method, an issue that could not be achieved with the A-star algorithm, so the latter was used to estimate the geometric tortuosity in media porous 40 × 40 × 40.

Finally, for better reproduction of the results obtained, correlations of the geometric tortuosity are established based on the porosity and the pore size distribution represented by the sigma generation parameter, in addition to leaving a correlation of the geometric tortuosity based on average pore size. These correlations were selected considering the Sum of squared errors and R-squared statistical parameters to guarantee the fitting. A possible advance in this study would be to calculate the geometric tortuosity using the pore network of the porous medium, also considering new approaches like artificial intelligence on 3D porous media generation.

## Supplementary Information

Supplementary Information.

## Data Availability

The datasets generated and/or analyzed during the current study are available from the corresponding author upon reasonable request.

## List of symbols

$$\mathrm{\varphi }$$: Porosity
$\sigma$: Sigma (standard deviation of Gaussian kernel)
$\updelta$: Blobiness or blobs
$$\tau$$: Tortuosity
IUPAC: International union of pure and applied chemistry
$${\tau }_{g}$$: Geometric tortuosity
A-star: A-star search algorithm
*C*: Variable to save path information
${\varepsilon }_{2D}$: Porosity two-dimensional
L: Length of the sample
$${L}_{g}$$: Geometric path
*m*: Maximum number for an iterative process
2D: Two-dimensional
3D: Three-dimensional
${p}_{in}$: Number of inlet nodes for pathfinding
${p}_{out}$: Number of outlet nodes for pathfinding
${n}_{in}$: Number of inlet nodes
${n}_{in2D}$: Number of inlet nodes for two-dimensional porous media
${n}_{in3D}$: Number of inlet nodes for three-dimensional porous media
$n$: Size in nodes per axis in porous media
$\rho$: Average pore size
SSE: Sum of squared errors of prediction
R-squared: Coefficient of determination R^2^
PSF: Pore size function of Porespy code
PDF: Probability density function
PSD: Pore size distribution
CDF: Cumulative distribution function
CSD: Cumulative standard deviation
SEM: Standard error of the mean
CV: Coefficient of variation
LLN: Law of large numbers
