# Supplementary material for: Evaluation of geometric tortuosity for 3D digitally generated porous media considering the pore size distribution and the A-star algorithm
Source: Sci Rep. 2022 Nov 14;12:19463. doi: 10.1038/s41598-022-23643-6 (PMC9663496; doi:10.1038/s41598-022-23643-6)
Supplement: Supplementary file 1 — Supplementary Information. [file 41598_2022_23643_MOESM1_ESM.docx]

**Appendix A. Determination of the number of samples**

To determine the number of geometric tortuosity samples required in the study, an analysis of the cumulative standard deviation (CSD) and the standard error of the mean (SEM) was performed based on [56]. Figure 22 shows the CSD behavior, which stabilizes around the sample number 50 to 60. It is important to notice that the CSD of the first 20 consecutive samples is less than 0.025, and the average calculated that determines the coefficient of variation (CV) for all models is 1.8868 %. Based on the SEM obtained for all samples, the real population falls between 4.319 and 1.120 with a 95% confidence level [56].

| 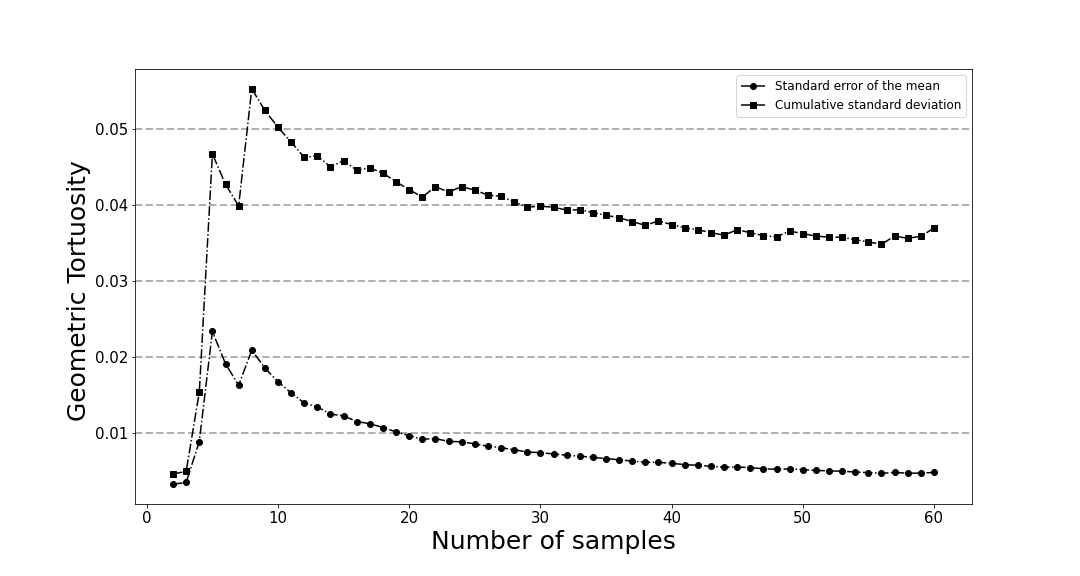 |
| --- |
| **Figure 22.** CSD and SEM behavior of geometric tortuosity of calculated samples |

In addition, the change in the cumulative average was analyzed as presented in Figure 23. The trend is that the cumulative average change decreases as the number of samples increases, which is compatible with the law of large numbers (LLN) in experiments [57]. The solid line approximates the behavior of the change curve in the cumulative mean when adding the next additional sample. A function to evaluate the tortuosity behavior can be expressed as follows:

| $f\left( n \right)=0.09337n^{-0.7463}$ | (10) |
| --- | --- |

| 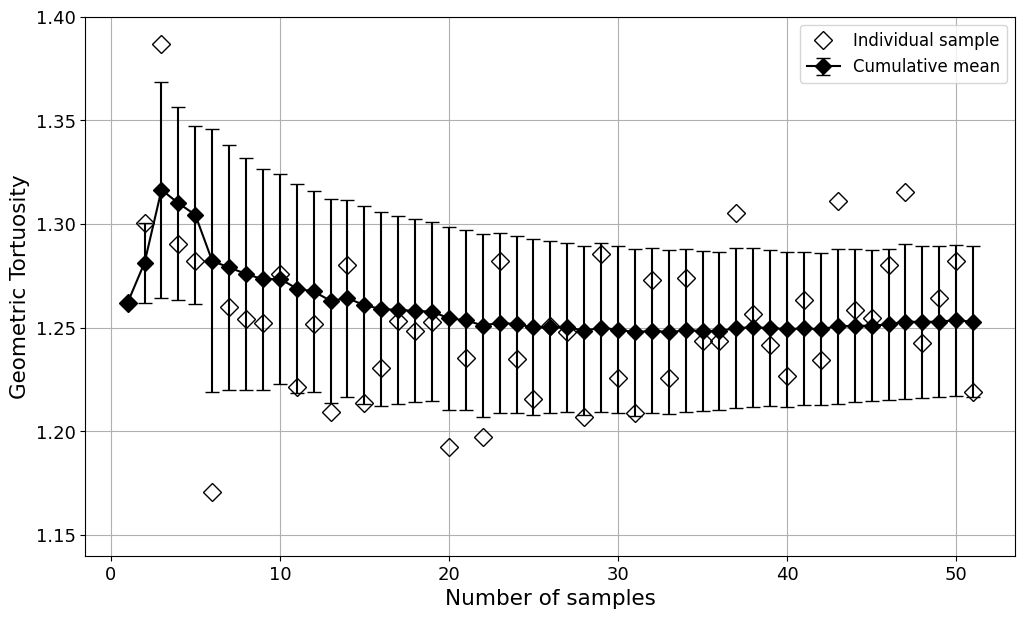 |
| --- |
| **Figure 23.** Tortuosity Geometric values were computed for each Porous Media sample with a porosity of 0.7. All the values fall in the range of 1.15 and 1.40. |

**Appendix B. Size independent Analysis**

One of the first attempts to evaluate the dependence on the size of the porous medium is to calculate the geometric tortuosity for the same porous media ($\phi=0.50)$ but different domain sizes. Although, at first glance, there is no change in the porous medium and geometric tortuosity, a deep analysis of tortuosity is needed. An investigation is performed with different size domains to evaluate the errors that can involve the size of the generated domains, as shown in Figure 24.

| 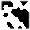 | 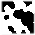 | 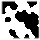 | 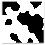 | 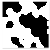 |
| --- | --- | --- | --- | --- |
| a) | b) | c) | d) | e) |
| **Figure 24.** 2D slices of samples with the same porosity but with different sizes: a) 30x30, b) 35x35, c) 40x40, d) 45x45, e) 50x50. | | | | |

The principle of this analysis is to guarantee that the study's algorithm is suitable for working with different media without worrying about the size or taking a specific standard value. Due to computational costs, an average sample is considered a reference, i.e., a 40x40x40 sample. The other sample sizes are tested with the same conditions in different dimensions. Therefore, this analysis is performed on media with the same porosity of 0.5 and sizes from 30x30x30 to 50x50x50, one sample for every size. Geometric tortuosity is computed to perform the comparison. As shown in Figure 25, the variations of the tortuosity values ​​do not exceed 5% concerning the reference. The maximum deviation is presented in the dimensions 50x50x50 with 3.32%. Therefore, it can be stated that the size does not cause a substantial impact on the tortuosity values.

| 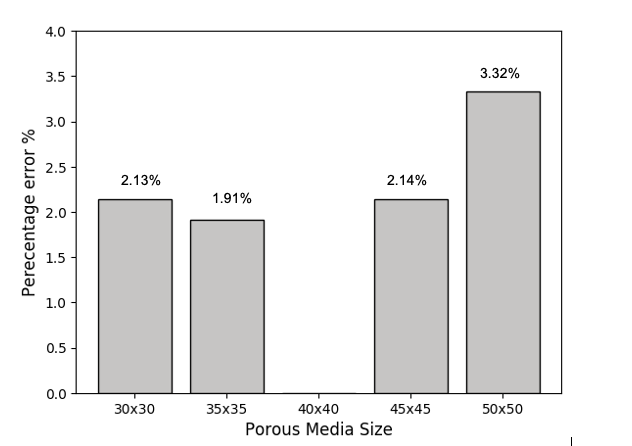 |
| --- |
| **Figure 25.** Tortuosity variation in porous media for different size domains taking as reference 40 side domain |

**Appendix C. Representative Elementary Volume**

As this is a microstructural analysis, the representative elemental volume (REV) should be analyzed. The REV corresponds to the minimum volume that can represent a whole porous medium. It is used to find out which sample may possess approximately the same properties as a larger sample. Therefore, it is applied by taking volume portions of a porous medium, in this case, a larger porous medium. The objective of this is to know if the dimension considered in the present study can represent a larger porous medium. The REV is usually estimated by changing the porosity [46], but can also be used for other microstructural parameters [8]. In this case, although the study deals with geometric tortuosity, the porosity is considered to estimate the REV due to its dependence already reviewed in the literature. As shown in Figure 26, a medium with a volume of 343000 voxels or dimensions of 70x70x70 with porosity 0.7 and blobiness 0.7 was considered, as these are the intermediate porosity and blobiness values of our study, to estimate the REV. The information that needs to be known is the approximate variability that could occur in porosity when studying a volume corresponding to the 40x40x40 medium, which would be 64000 voxels. If we look at the graph, the variability of most of the data from volume 64000 is less than 0.05 in porosity. Of course, this variation is reduced as it approaches a volume similar to the complete medium. Although this error is not so small, it can be considered acceptable for the present study. Therefore, it can be stated that the present porous media size of 40x40x40 can satisfy the REV of 70x70x70 porous media.

| **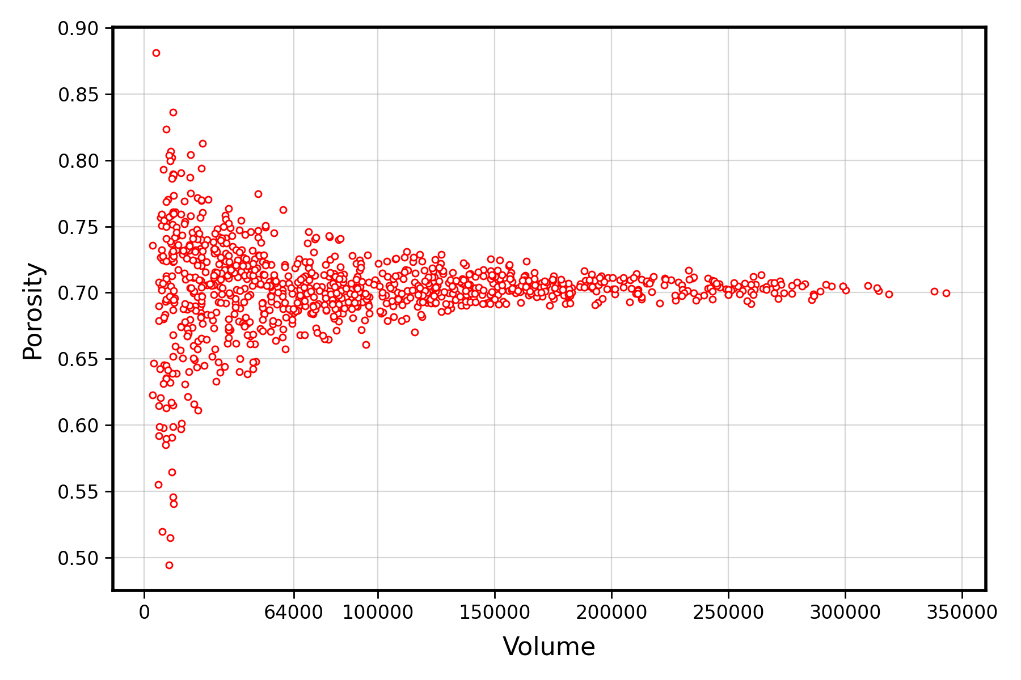** |
| --- |
| **Figure 26.** Representative elementary volume of a 70x70x70 porous media |

**Appendix D. A-start Pseudocode**

| Initialize OPEN list  Initialize CLOSED list  Create arrival node; called node_goal (or endpoint)  Create starting node; called node_start (or starting point)  Add the node_start to the OPEN list  While the OPEN list is not empty {  Get node (x,y,z) out of the OPEN list with the lowest f (x, y, z)  Add (x, y, z) to the CLOSED list  If (x, y, z) is the same as node_goal, we have found the solution; return Solution (x, y, z)  Generate each successor node (x, y, z)' of (x, y, z)  for each successor node (x, y, z)' of (x, y, z) {  Set the parent of (x, y, z)' to (x, y, z)'  Set h ((x, y, z)') to be the heuristically estimated distance to node_goal  Set g ((x, y, z)') to g ((x, y, z)) plus the cost to get to (x, y, z)' from (x, y, z)  Set f ((x, y, z)') to g ((x, y, z)') plus h ((x, y, z)')  if (x, y, z) 'is in the OPEN list and the existing one is as good or better, then discard (x, y, z)' and continue  if (x, y, z) 'is in the CLOSED list and the existing one is as good or better, then discard  (x, y, z)' and continues  Eliminate occurrences of (x, y, z)' of OPEN and CLOSED  Add (x, y, z) 'to the OPEN list  }  }  Return error (If we get to this point, we have searched all reachable nodes and have not found the solution yet; therefore, it does not exist) |
| --- |
|  |
